# Impaired hematopoiesis affects apheresis and CAR T‐cell product composition and treatment response

**DOI:** 10.1111/trf.70224

**Published:** 2026-04-10

**Authors:** Hanna Kuhn, Felix Korell, Angela Hückelhoven‐Krauss, Brigitte Neuber, Miriam Stenzinger, Anita Ludwig‐Husemann, Maria‐Luisa Schubert, Patrick Derigs, Kiavasch Farid, Tim Sauer, Sandra Sauer, Carsten Müller‐Tidow, Peter Dreger, Michael Schmitt, Anita Schmitt

**Affiliations:** ^1^ Internal Medicine V, Hematology, Oncology and Rheumatology Heidelberg University Hospital Heidelberg Germany; ^2^ Institute of Clinical Transfusion Medicine and Cell Therapy (IKTZ) Heidelberg Germany; ^3^ Institute of Immunology University Hospital Heidelberg Heidelberg Germany

## Abstract

**Background:**

Chimeric antigen receptor (CAR) T‐cell therapy has transformed the treatment of hematologic malignancies, but its success depends on obtaining sufficient CD3^+^ T‐cell yields during leukapheresis. This can be difficult in heavily pretreated patients, who often show leukopenia and reduced T‐cell fitness.

**Methods:**

We analyzed 166 leukapheresis products from 154 patients undergoing manufacturing of CD19‐directed CAR T‐cells, including 146 from non‐Hodgkin's lymphoma (NHL) and 20 from acute lymphoblastic leukemia (ALL). Collections were performed for commercial CAR T‐cell products (axi‐cel, tisa‐cel, brexu‐cel, liso‐cel; *n* = 121) and the HD‐CAR‐1 trial with in‐house manufacturing (heidagenlecleucel; *n* = 45).

**Results:**

In 150/154 patients, a sufficient CD3 ^+^ T‐cell yield was achieved by a single leukapheresis. Pre‐apheresis lymphocyte count strongly predicted CD3 ^+^ T‐cell yield (*p*< .001) and was associated with treatment response (*p* = .044). Impaired hematopoiesis, reflected by reduced nucleated cell count (*p*< .001), lymphocyte count (*p* <.001), and hematocrit (*p* = .017), was linked to poorer collection efficiency. Intensive prior therapies, including stem cell transplantation, reduced CAR T‐cell expansion during manufacturing (*p* = .036). In patients with ALL, higher proportions of effector and CD8 ^+^ CAR T‐cells in the product correlated with improved clinical outcomes (*p* = .04; *p* = .023).

**Conclusion:**

These findings highlight the importance of early leukapheresis, ideally before intensive treatments, to optimize T‐cell yield, product quality, and therapeutic efficacy.

**Abbreviations**
ACD‐A: acid citrate dextrose solution A
ALL: acute lymphoblastic leukemia
Allo: allogeneic
Auto: autologous
Axi‐cel: axicabtagene ciloleucel
B‐PLL: B‐cell prolymphocytic leukemia
Brexu‐cel: brexucabtagene autoleucel
BSA: body surface area
C: cyclophosphamide
CAR: chimeric antigen receptor
CLL: chronic lymphocytic leukemia
CR: complete response
CRS: cytokine release syndrome
DLBCL: diffuse large B‐cell lymphoma
EMA: European Medicines Agency
f: female
F: fludarabine
FDA: U.S. Food and Drug Administration
FL: follicular lymphoma
FL3B: follicular lymphoma grade 3b
Hb: hemoglobin
HBV: hepatitis B virus
Hct: Hematocrit
ICANS: immune effector cell‐associated neurotoxicity syndrome
LBCL: large B‐cell lymphoma
Liso‐cel: lisocabtagene maraleucel
m: male
MAS: macrophage activation syndrome
MCL: mantle cell lymphoma
MRD: minimal residual disease
NC: nucleated cells
NE: not evaluable
NHL: Non‐Hodgkin Lymphoma
NI: no infusion
PD: progressive disease
Ped: pediatric
PMBCL: primary mediastinal B‐cell lymphoma
PR: partial response
r/r: relapsed/refractory
SCT: stem cell transplantation
SD: stable disease
TBV: total blood volume
TCM: central memory T‐cells
TEFF: effector T‐cells
TEM: effector memory T‐cells
Tisa‐cel: tisagenlecleucel
TN: naïve T‐cells
TNC: total nucleated cells
Tx: therapy
WBC: white blood cells
Y/o: years old

## 1 INTRODUCTION

Since its US Food and Drug Administration (FDA) approval in 2017 for relapsed/refractory (r/r) B‐cell malignancies, chimeric antigen receptor (CAR) T‐cell therapy has transformed cancer treatment, demonstrating remarkable clinical success.^1^ Nevertheless, long‐term disease control is achieved in fewer than 50% of patients with large B‐cell lymphoma (LBCL),^2^, ^3^, ^4^ whereas higher rates of durable disease control have been reported in patients with indolent non‐Hodgkin Lymphoma (NHL).^5^, ^6^ Factors limiting durable responses include tumor antigen escape, poor CAR T‐cell trafficking and infiltration, an immunosuppressive microenvironment, and the immunophenotype of the CAR T‐cells in the product.^7^

CAR T‐cell therapy begins with autologous T‐cell collection via unstimulated leukapheresis. Eligible patients are often heavily pretreated and exhibit impaired hematopoiesis, which can reduce peripheral lymphocyte counts and limit both the yield and quality of collected T‐cells. Impaired hematopoiesis may not only hinder manufacturing feasibility but also impact therapy efficacy, as higher T‐cell counts in the apheresis correlate with improved clinical responses.^8^ Prior cytotoxic therapies are associated with terminally differentiated or exhausted T‐cell phenotypes, reduced naïve and early memory subsets, and diminished proliferative potential, all of which compromise CAR T‐cell manufacturing and anti‐tumor efficacy.^9^, ^10^, ^11^, ^12^, ^13^ Current guidelines recommend minimum wash‐out periods and thresholds for hematologic recovery, including absolute lymphocyte and white blood cell counts, yet these metrics may not fully reflect functional immune reconstitution or predict apheresis success. The composition of the final CAR T‐cell product, beyond total T‐cell number, strongly influences outcomes. Some studies emphasize the importance of specific T‐cell subsets, while others suggest that the CD4^+^/CD8^+^ ratio is critical for therapeutic efficacy^14^, ^15^, ^16^ with some trials infusing defined ratios to enhance therapeutic success.^17^, ^18^ In line with this strategy, lisocabtagene maraleucel was approved by the FDA in 2021 and the European Medicines Agency (EMA) in 2022 as a CAR T‐cell product with a fixed 1:1 CD4^+^:CD8^+^ T‐cell composition.

These findings highlight the interplay between hematopoietic integrity, leukapheresis success, T‐cell subset composition, and CAR T‐cell efficacy. As CAR T‐cell therapy is discussed to expand into earlier treatment lines^19^, ^20^, ^21^, ^22^ and potentially solid tumors,^23^ understanding the factors that influence T‐cell collection and product quality is essential for optimizing outcomes. In this study, we aimed to correlate pre‐apheresis lymphocyte count with CD3^+^ T‐cell yield and treatment response and to evaluate the impact of CD8^+^ and effector T‐cells on the outcomes of patients with acute lymphoblastic leukemia (ALL) treated with HD‐CAR‐19T‐cells.

## 2 MATERIALS AND METHODS

### 2.1 Patients

In this study, we assessed 154 patients undergoing leukapheresis for CAR T‐cell production at Heidelberg University Hospital from September 2018 to May 2023. A second apheresis session was required for 12 patients, resulting in a total of 166 analyzed apheresis procedures. Overall, five different CAR T‐cell products were administered during this study, including the CD19‐directed CAR T‐cell products axicabtagene ciloleucel (axi‐cel) and brexucabtagene autoleucel (brexu‐cel) from Gilead Sciences/Kite Pharma (Santa Monica, CA, USA); tisagenlecleucel (tisa‐cel) from Novartis (Basel, Switzerland); lisocabtagene maraleucel (liso‐cel) from Bristol Myers Squibb (New York City, NY, USA); and in‐house produced CD19‐specific third‐generation CAR T‐cells (heidagenlecleucel, HD‐CAR‐19) as part of the HD‐CAR‐1 trial (Heidelberg, Germany).

### 2.2 Leukapheresis procedures

Leukapheresis procedures were conducted at Heidelberg University Hospital in collaboration with the Institute of Clinical Transfusion Medicine and Cell Therapy (IKTZ, Heidelberg, Germany), using the Spectra Optia apheresis system (Terumo, Tokyo, Japan).

In this study 166 unstimulated leukaphereses were investigated in patients with diffuse large B‐cell lymphoma (DLBCL, *n* = 110), chronic lymphocytic leukemia (CLL, *n* = 11), mantle cell lymphoma (MCL, *n* = 16), follicular lymphoma (FL, *n* = 6), primary mediastinal B‐cell lymphoma (PMBCL, *n* = 2), B‐cell prolymphocytic leukemia (B‐PLL, *n* = 1), and ALL, *n* = 20. Apheresis sessions lasted between 2 and 5 h, processing the patient's total blood volume (TBV) 2–4 times. The TBV was calculated according to the Nadler formula. For anticoagulation, acid citrate dextrose solution A (ACD‐A) was used at a blood‐to‐anticoagulant ratio of 10–14:1. Collection procedures were usually performed through peripheral veins using ultrasound‐guided puncture when venous access was difficult, whereas central venous access was necessary only in a few cases. Supplementary Table 1 outlines indications and requirements for each CAR T‐cell product.

### 2.3 Leukapheresis products

Flow cytometry analysis of the aphereses were conducted at the IKTZ. Viability analysis using propidium iodide, along with the assessment of CD3^+^/CD45^+^ T‐cells and total nucleated cell (NC) count, was performed with the MACSQuant‐Analyzer 10 (Miltenyi Biotech, Bergisch Gladbach, Germany). Staining was performed using CD3‐PE (Cat. N. 345,765) and CD45‐FITC (Cat. N. 345,808) antibodies from Becton Dickinson (Franklin Lakes, NJ, USA). Hematocrit (hct) and platelet counts were measured using an automated hematology analyzer (XP‐300, Sysmex, Norderstedt, Germany). The sterility of the leukapheresis product was ensured using BACTEC™ flasks from Becton Dickinson for automated blood culture testing (Hybeta, Germany) and BacT/ALERT™ (bioMérieux, Marcy‐l'Étoile, France) at the German Red Cross blood donation center in Mannheim, Germany. The tisa‐cel leukapheresis product was cryopreserved according to the CAR‐T cell manufacturer's specifications at the IKTZ. For the axi‐cel, brexu‐cel, and liso‐cel products, the leukapheresis was collected freshly on the same day for centralized cryopreservation. For the HD‐CAR‐1 study, the fresh leukapheresis product was cryopreserved at the GMP Core Facility of the Heidelberg University Hospital.

### 2.4 T‐cell immunophenotyping in the HD‐CAR‐19 cohort

HD‐CAR‐19 apheresis and final CAR T‐cell product composition was determined by flow cytometry using the LSRFortessa II or the FACSCanto from Becton Dickinson. Staining was performed with the following antibodies: CD3‐FITC (Cat. N. 345763), CD4‐APC (Cat. N. 345771), CD8‐PE‐Cy7 (Cat. N. 335822), CD19‐APC (Cat. N. 345791) and CD45‐PerCP (Cat. N. 345809) from Becton Dickinson; CD45RA‐APC (Cat. N. 17‐0458‐42) from eBioscience (San Diego, CA, USA) and CD197‐PerCP (Cat. N. 353242) from Biolegend (San Diego, CA, USA). Lymphocytes were defined as B‐lymphocytes: CD45^+^, CD19^+^ or T‐lymphocytes: CD45^+^ CD3^+^. T‐cell subsets were defined as followed: naïve T‐cells (T_N_): CD45RA^+^ CCR7^+^; effector T‐cells (T_EFF_): CD45RA^+^ CCR7^−^; central memory T‐cells (T_CM_): CD45RA^−^ CCR7^+^; effector memory T‐cells (T_EM_): CD45RA^−^ CCR7^−^; and as CD4^+^, CD8^+^, CD8^−^CD4^−^, and CD8^+^CD4^+^ T‐cells (Supplementary Figure 1).

### 2.5 Clinical evaluation of best response and CRS/ICANS

The clinical response to CAR T‐cell therapy was evaluated based on the Lugano standard response criteria for NHL, categorizing outcomes as complete remission (CR), partial remission (PR), stable disease (SD), progressive disease (PD), and not yet evaluable (NE).^24^ For ALL, the assessment also included blast‐percentage and minimal residual disease testing.^25^ The severity of both Cytokine Release Syndrome (CRS) and Immune Effector Cell‐Associated Neurotoxicity Syndrome (ICANS) was graded using the criteria established by Lee et al.^26^ (ZUMA‐1) and the American Society for Transplantation and Cellular Therapy consensus criteria (validation cohort).^27^, ^28^

### 2.6 Data analysis

Data were collected using Microsoft Excel 16.87 for Mac (Microsoft Corporation, Redmond, WA, USA) and analyzed with IBM SPSS 30 for Mac (IBM Corporation, Armonk, NY, USA). Spearman's rank correlation assessed associations between continuous variables. For two‐group comparisons, Student's *t‐*test was used when variance homogeneity (Levene's test) assumptions were met; otherwise, Welch's *t*‐test was applied. The Kruskal–Wallis test with Bonferroni correction was used for comparisons involving more than two groups without assuming normality. Differences in CRS and ICANS incidence across CAR T‐cell products were analyzed using the Chi‐square test. Statistical significance was set at *p* <.05. Figures were created with BioRender.com.

## 3 RESULTS

### 3.1 Patients' characteristics

Among 154 patients with hematologic malignancies—including DLBCL, CLL, MCL, FL, PMBCL, ALL, and B‐PLL—apheresis was performed to produce CAR T‐cells, including commercial products (axi‐cel, tisa‐cel, brexu‐cel, liso‐cel) or in‐house produced HD‐CAR‐19T‐cells (Table 1). HD‐CAR‐19T‐cells were administered in six dose levels (1–200 × 10^6^) (Supplementary Table 2).

**TABLE 1:** Patients' characteristics.

CD19.CAR T‐cell products	*n* patients undergoing apheresis, gender	Age, year (median, range)	Weight, kg (median, range)	Height, cm (median, range)	TBV, L (median, range)	Disease	Prior therapy lines (median, range)	Best response
Axi‐cel (Yescarta)	67 (17 f, 50 m)	57 (21–77)	79 (46–147)	178 (152–193)	5.2 (3.4–7.5)	64 DLBCL (96%), 1 FL (1%), 2 PMBCL (3%)	4 (2–9)	23 CR (34%), 24 PR (36%), 7 SD (10%), 4 PD (6%), 1 NE (1%), 8 NI (12%)
Tisa‐cel (Kymriah)	27 (5 f, 22 m)	70 (21–83)	71 (50–102)	176 (156–186)	4.9 (3.4–5.9)	23 DLBCL (85%), 2 FL (7%), 2 ALL (7%)	4 (2–6)	3 CR (11%), 5 PR (19%), 7 SD (26%), 2 PD (7%), 3 NE (11%), 7 NI (26%)
Brexu‐cel (Tecartus)	13 (4 f, 9 m)	66 (57–77)	70 (48–110)	177 (155–193)	4.9 (3.3–6.6)	2 DLBCL (15%), 11 MCL (85%)	4 (3–6)	7 CR (54%), 1 PR (8%), 3 SD (23%), 1 NE (8%), 1 NI (8%)
Liso‐cel (Breyanzi)	4 (1 f, 3 m)	77 (73–83)	73 (56–93)	175 (162–178)	4.9 (3.5–5.7)	4 DLBCL (100%)	3 (2–6)	3 CR (75%), 1 NI (25%)
HD‐CAR‐19	43 (17 f, 26 m)	58 (21–77)	78 (47–160)	175 (150–197)	4.8 (3.2–8.4)	7 DLBCL (16%), 9 CLL (21%), 5 MCL (12%), 3 FL (7%), 18 ALL (42%), 1 B‐PLL (2%)	5 (2–11)	21 CR (49%), 4 PR (9%), 5 SD (12%), 8 PD (19%), 1 NE (2%), 3 NI (7%)

Abbreviations: ALL, acute lymphocytic leukemia; B‐PLL, B‐cell prolymphocytic leukemia; CLL, chronic lymphocytic leukemia; CR, complete response; DLBCL, diffuse large B‐cell lymphoma; f, female; FL, follicular lymphoma; m, male; MCL, mantle cell lymphoma; NE; not evaluated; NI, no infusion; PD, progressive disease; PMBCL, primary mediastinal B‐cell lymphoma; PR, partial response; SD, stable disease; TBV, total blood volume.

### 3.2 Leukapheresis procedures and product composition

The median lymphocyte count prior to apheresis was 0.76/nL (0.03–7.55/nL). Throughout apheresis, the median blood volume managed was 12 L (5.1–20.9 L) with the TBV being processed 2.5 (1.2–4) times, resulting in a median duration of 248 min (120–326 min) of the apheresis procedure (Table 2).

**TABLE 2:** Leukapheresis Procedures.

Diagnosis	Apheresis products, *n*	Leukapheresis duration, min (median, range)	Processed blood volume, L (median, range)	Times TBV processed (median, range)	Lymphocyte count prior/nL (median, range)
All patients	166	248 (120–326)	12 (5.1–20.9)	2.5 (1.2–4)	0.76 (0.03–7.55)
DLBCL	110	249 (120–325)	12.1 (6.5–20.9)	2.5 (1.3–4)	0.63 (0.11–3.38)
CLL	11	193 (126–305)	10 (8.6–14.1)	1.9 (1.3–3.2)	1.09 (0.42–7.55)
MCL	16	235 (165–298)	12.8 (7.3–15)	2.3 (1.2–3.7)	0.97 (0.11–4.5)
FL	6	280 (140–300)	14.8 (5.8–15.4)	2.3 (1.5–3.9)	0.94 (0.67–2)
PMBCL	2	257 (248–266)	12	2.5 (2.3–2.6)	0.46 (0.38–0.54)
B‐PLL	1	292	13.9	2.9	0.99
ALL	20	248 (138–326)	8.9 (5.1–15)	1.9 (1.3–3.1)	1.2 (0.03–2.18)

Abbreviations: ALL, acute lymphocytic leukemia; B‐PLL, B‐cell prolymphocytic leukemia; CLL, chronic lymphocytic leukemia; DLBCL, diffuse large B‐cell lymphoma; FL, follicular lymphoma; MCL, mantle cell lymphoma; PMBCL, primary mediastinal B‐cell lymphoma; TBV, total blood volume.

Apheresis product composition did not differ significantly among disease subgroups. Overall, the apheresis products contained a median of 43.3 × 10^8^ (4.0–231.8 × 10^8^) CD3^+^ T‐cells, 111.4 × 10^8^ (9.3–709.8 × 10^8^) NCs, and 1136 × 10^6^/mL (32–4958 × 10^6^/mL) platelets. The median hematocrit was 2.0% (0.8–7.4%) and the viability was 99.9% (99.2%–100%) (Table 3).

**TABLE 3:** Leukapheresis characteristics.

Diagnosis	Apheresis products, *n*	CD3^+^, ×10^8^ (median, range)	NC, ×10^8^ (median, range)	Volume, mL (median, range)	Hematocrit, % (median, range)	Platelets, ×10^6^/mL (median, range)	CAR T‐cell products
All patients	166 (46 f, 120 m)	43.3 (4.0–231.8)	111.4 (9.3–709.8)	250 (127–349)	2.0 (0.8–7.4)	1136 (32–4958)	73 Axi‐cel, 31 Tisa‐cel, 45 HD‐CAR‐19, 13 Brexu‐cel, 4 Liso‐cel
DLBCL	110 (30 f, 80 m)	43.3 (4.2–231.8)	115.5 (9.3–340.5)	254 (136–347)	1.9 (0.8–7.4)	1173 (147–4958)	70 Axi‐cel, 27 Tisa‐cel, 7 HD‐CAR‐19, 2 Brexu‐cel, 4 Liso‐cel
CLL	11 (3 f, 8 m)	39.6 (6.4–99.7)	101.5 (14.8–709.8)	212 (152–299)	1.7 (1.4–5.7)	100 (845–2845)	11 HD‐CAR‐19
MCL	16 (2 f, 14 m)	50.4 (16–106.6)	125.6 (72.1–223)	244 (182–349)	1.9 (1.1–4.6)	1394 (742–3120)	5 HD‐CAR‐19, 11 Brexu‐cel
FL	6 (2 f, 4 m)	46.7 (36.8–131.4)	115.3 (54.9–241.3)	265 (165–302)	2.0 (1.1–2.9)	1365 (164–2500)	1 Axi‐cel, 2 Tisa‐cel, 3 HD‐CAR‐19
PMBCL	2 (2 m)	21.3 (17.7–24.9)	65.2 (58.8–71.5)	262 (260–263)	5.5 (3.9–7.1)	1125 (1016–1234)	2 Axi‐cel
B‐PLL	1 (1 m)	100.3	169.9	304	1.7	1190	1 HD‐CAR‐19
ALL	20 (9 f, 11 m)	27.0 (4.0–150.4)	85.2 (19.7–296.0)	236 (127–313)	2.0 (0.8–5.1)	628 (32–3045)	2 Tisa‐cel, 18 HD‐CAR‐19

Abbreviations: ALL, acute lymphocytic leukemia; B‐PLL, B‐cell prolymphocytic leukemia; CLL, chronic lymphocytic leukemia; DLBCL, diffuse large B‐cell lymphoma; f, female; FL, follicular lymphoma; m, male; MCL, mantle cell lymphoma; NC, nucleated cells; PMBCL, primary mediastinal B‐cell lymphoma.

A second apheresis was necessary for 12 patients (Supplementary Table 3). Among them, three patients undergoing apheresis for tisa‐cel production initially failed to reach the target yield of ≥10 × 10^8^ CD3^+^ T‐cells but achieved the cumulative target on the following day. Additionally, one patient scheduled for axi‐cel production exhibited a markedly low CD3^+^ T‐cell yield, possibly due to prior treatment with Polatuzumab vedotin and Bendamustine three weeks before apheresis.

### 3.3 Pre‐apheresis lymphocyte count and CD3 ^+^ T‐cell yield

**FIGURE 1:**
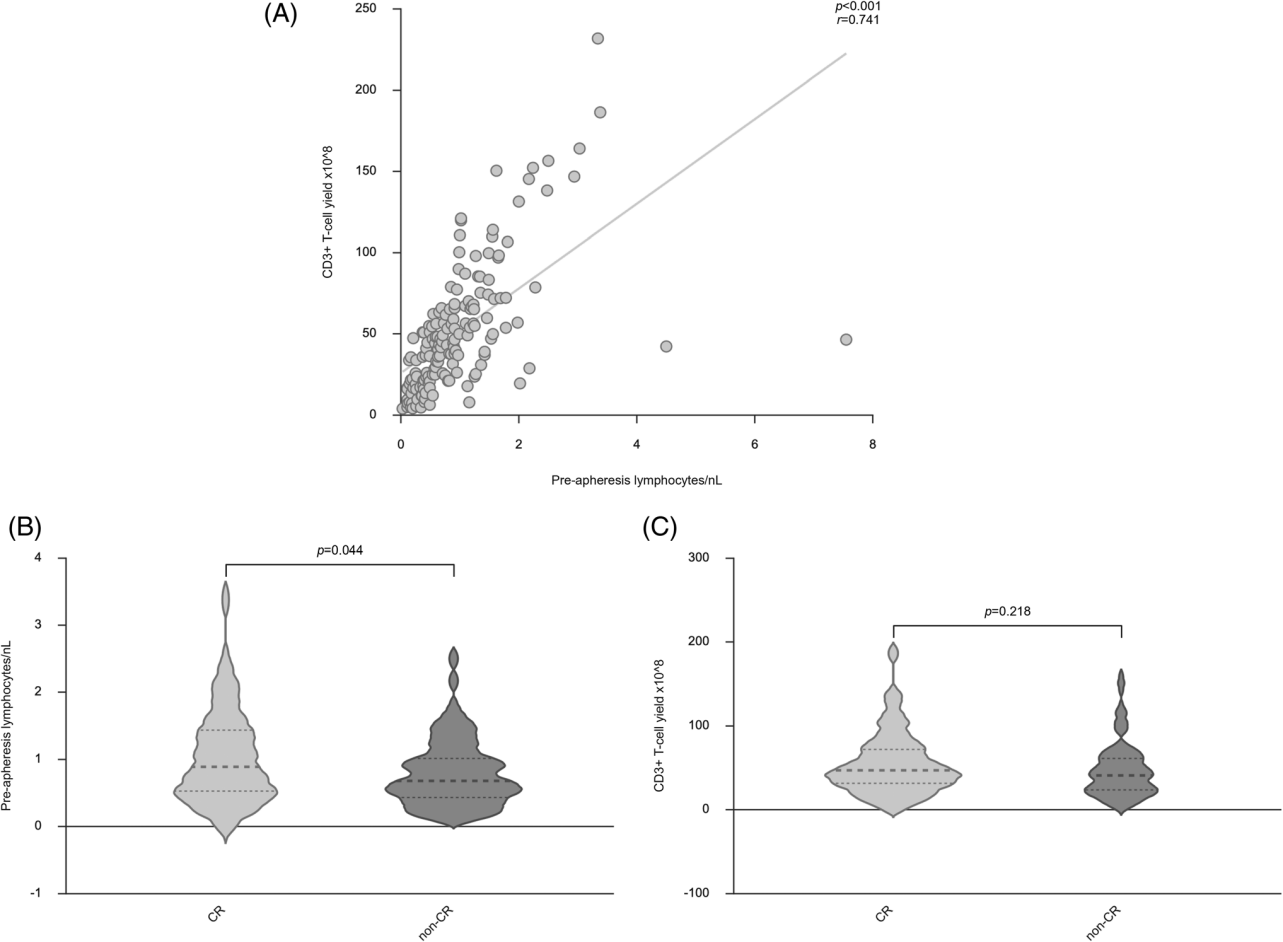
Correlation of lymphocyte count pre‐apheresis with CD3^+^ T‐cell yield and treatment response. (A) Analysis of CD3^+^ T‐cell yield by lymphocyte count pre‐apheresis (*n* = 164). *R*‐ and *p*‐values were calculated using Spearman's correlation. Comparing (B) pre‐apheresis lymphocyte count/nL (*n* = 126) and (C) CD3^+^ T‐cell yield × 10^8^ (*n* = 127) between patients with CR and patients with PR, SD, and PD (non‐CR). *p*‐values were calculated using Welch's test for (B) and student's *t*‐test for (C).

Lymphocyte count prior to leukapheresis correlated strongly with CD3^+^ T‐cell yield (*r* = 0.741, *p* <.001) (Figure 1A). Additionally, CD3^+^ T‐cell yield was significantly associated with an increased NC count (*r* = 0.334, *p* <.001) and Hct (*r* = 0.186, *p* = .017) pre‐apheresis, while the correlation with hemoglobin (hb) (*r* = 0.134, *p* = .087) showed only a non‐significant trend (Supplementary Figure 2A–C). Even in patients with a low lymphocyte count as low as 0.11/nL, an adequate CD3^+^ T‐cell yield followed by successful CAR T‐cell manufacturing was achieved. For one patient with ALL in the HD‐CAR‐1 trial with a lymphocyte count of 0.03/nL, a CAR T‐cell product for dose level 1 (1 × 10^6^ CAR T‐cells/m^2^) could be manufactured successfully. Overall, 37.9% of the patients had a very low lymphocyte count below 0.6/nL, 29.4% had a low lymphocyte count of 0.6–1/nL, leaving only 32.7% of the patients with a lymphocyte count above 1/nL before apheresis. Interestingly, a higher lymphocyte count pre‐apheresis but not CD3^+^ T‐cell yield was associated with a better treatment response (*p* = .044) (Figure 1B,C).

### 3.4 T‐cell immunophenotyping in the HD‐CAR‐19 cohort

T‐cell subset composition of apheresis and CAR T‐cell products in the HD‐CAR‐19 cohort was analyzed for CD4^+^/CD8^+^ ratio and proportion of T_N_, T_EFF_, T_CM_, and T_EM_.

**FIGURE 2:**
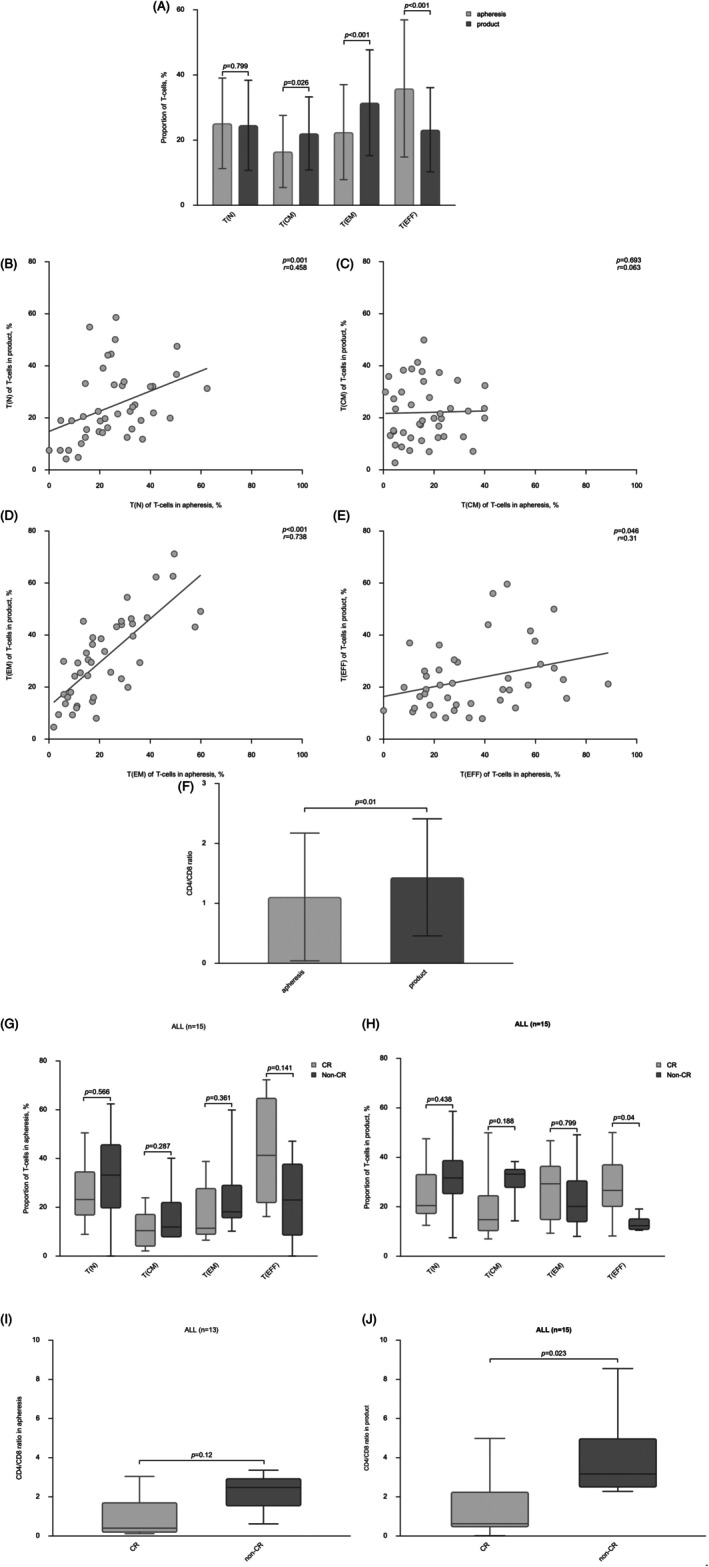
T‐cell immunophenotypes and CD4^+^/CD8^+^ ratio in the HD‐CAR‐19 cohort. (A) Comparison of T‐cell subsets between apheresis and product (*n* = 42). *p*‐values were calculated using paired *t*‐test. Data for comparison between two groups are represented as mean ± standard deviation (SD). (B)–(E) Correlation between T‐cell phenotypes in apheresis and product. Spearman's correlation was used for calculating *R*‐ and *p*‐values. (F) CD4^+^/CD8^+^ ratio in apheresis and product (*n* = 38). *p*‐values were calculated using paired student's *t*‐test. Data for comparison between two groups are represented as mean ± SD. (G), (H) Analysis of T‐cell immunophenotypes in apheresis and product of patients with ALL (*n* = 15) by response (CR vs. non‐CR). *p*‐values were calculated using student's *t*‐test. (I), (J) Evaluation of CD4^+^/CD8^+^ ratio in apheresis and product of patients with ALL (*n* = 13, 15) by response. *p*‐values were calculated using student's *t*‐test. Data in (G)–(J) is represented as a box plot.

Proportion of T‐cell subsets in both apheresis and final product varied strongly in between patients independent of their underlying diseases (Supplementary Figure 3). Overlooking all patients of the HD‐CAR‐19 cohort, T_EFF_ cells were more abundant in the apheresis concentrate than T_EM_ and T_CM_ subsets. The percentage of effector T‐cells decreased from 35.9% ± 21.1% in the apheresis to 23.2% ± 12.9% (*p* <.001) in the final product. The percentage of memory T‐cells increased from 22.4% ± 14.6% for T_EM_ and 16.5% ± 11.1% for T_CM_ in the apheresis product to 31.5% ± 16.2% for T_EM_ (*p* <.001) and 22.1% ± 11.2% for T_CM_ (*p* = .026) in the final product (Figure 2A). A higher proportion of T_N_ (*r* = 0.458, *p* = .001), T_EFF_ (*r* = 0.31, *p* = .046), and T_EM_ cells (*r* = 0.738, *p* <.001) in the apheresis product was strongly associated with a higher proportion of these subsets in the final product (Figure 2B–E).

Analyzing the CD4^+^/CD8^+^ ratio in both apheresis and CAR T‐cell product, the ratio increased from 1.11 ± 1.07 to 1.43 ± 0.98 in the final product (*p* = .01) (Figure 2F).

Fold expansion of T‐cells was determined from transduction to cryopreservation on day 10 or 13. T‐cell expansion was associated with a trend toward higher percentages of T_N_ cells (*r* = 0.276, *p* = .081) and a significantly reduced proportion of T_EFF_ cells (*r* = −0.342, *p* = .027) in the starting material for CAR T‐cell production. Stronger T‐cell proliferation also correlated with increased proportions of T_EM_ cells (*r* = 0.338, *p* = .028) in the final CAR T‐cell product. Notably, T‐cell proliferation was not associated with the CD4^+^/CD8^+^ T‐cell ratio in neither apheresis nor infusion product (Supplementary Figure 4).

Also, we compared T‐cell subset composition between patients with ALL (*n* = 15) and CR and patients with ALL and PR, SD or PD (= non‐CR patients). Interestingly, patients with CR had a significantly larger proportion of T_EFF_ cells (*p* = .04) and a lower CD4^+^/CD8^+^ ratio in their CAR T‐cell products (*p* = .023) (Figure 2G–J). For the second‐largest group—patients with CLL (*n* = 8)—no significant differences in T‐cell subset composition were observed between responders and non‐responders (Supplementary Figure 5).

### 3.5 Toxicity

**FIGURE 3:**
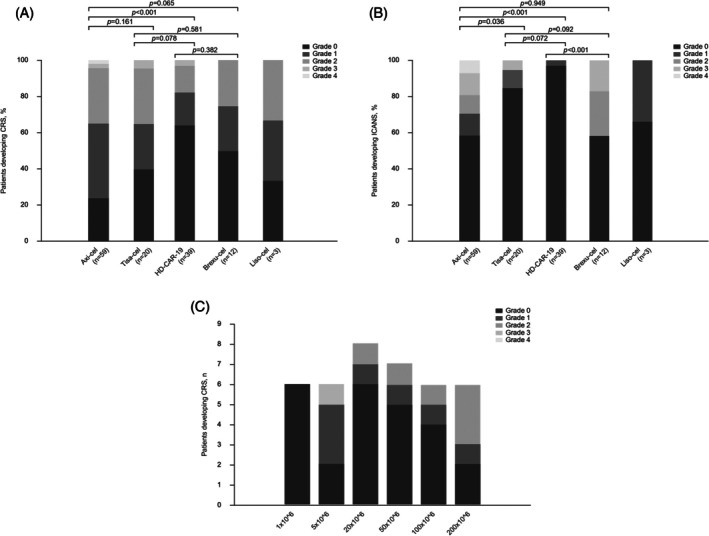
Occurrence of CRS and ICANS in patients receiving different CAR T‐cell products. Analysis of the occurrence and severity of CRS (A) and ICANS (B) by CAR T‐cell product. *p*‐values were calculated using Chi‐square test for CAR T‐cell products with *n* >10 total administrations. (C) Evaluation of the occurrence and severity of CRS in patients receiving HD‐CAR‐19T‐cells by the applied dose level.

The incidence of CRS and ICANS varied across CAR T‐cell products, with HD‐CAR‐19 showing very limited toxicity (Figure 3A,B). Of 40 patients within the HD‐CAR‐19 cohort, 39 were evaluable: 36% (14/39) developed CRS, mostly grade 1/2, except one grade 3 case. Even at the highest dose (200 × 10^6^ CAR T‐cells/m^2^), CRS remained grade 1/2, indicating no clear dose dependency (Figure 3C). ICANS was rare, with only one patient experiencing grade 1. Overall, 3 of 154 patients receiving CAR T‐cells experienced severe CRS or ICANS (grade ≥3) and died within 10–41 days post‐infusion, due to circulatory shock with macrophage activation syndrome or sepsis (Supplementary Table 4).

### 3.6 Impact of prior therapy lines

In patients with NHL, a higher number of prior therapy lines (>3) was associated with signs of impaired hematopoiesis, reflected by significantly lower platelet (*r* = −0.267, *p* = .03) and NC counts (*r* = −0.187, *p* = .03), including reduced granulocyte (*r* = −0.192, *p* = .026) and monocyte (*r* = −0.188, *p* = .03) counts in the peripheral blood pre‐apheresis. Additionally, patients with more prior therapy lines showed a trend toward lower NC (*r* = −0.151, *p* = .082) and platelet counts (*r* = −0.177, *p* = .053) in the apheresis product itself (Supplementary Figure 6). These findings highlight that hematopoietic suppression in heavily pre‐treated patients can adversely affect the apheresis product.

**FIGURE 4:**
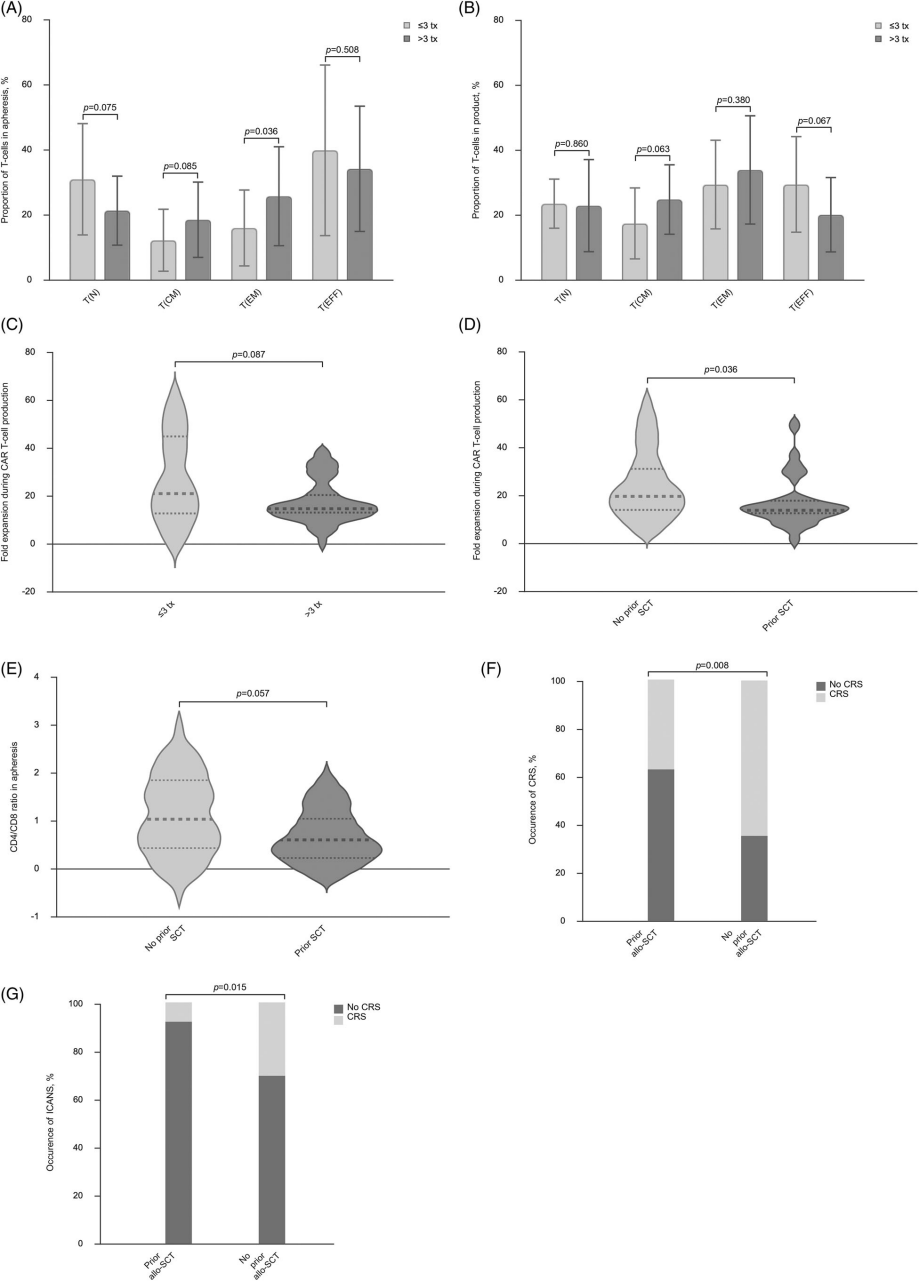
Impact of prior lines of therapy and SCT on T‐cell subset composition and fold expansion. (A), (B) Analysis of T‐cell immunophenotypes in apheresis and products by number of prior therapy lines (*n* = 40). *p*‐values were calculated using Welch‐test. Results are shown as mean ± SD. (C) Comparison of fold expansion ex vivo between patients with ≤3 and >3 previous lines of therapy (*n* = 40). *p*‐values were calculated using Welch‐test. (D) Fold expansion ex vivo in patients with prior SCT compared to patients without prior SCT (*n* = 42). Student's *t*‐test was used to calculate *p*‐values. (E) Analysis of CD4^+^/CD8^+^ ratio in apheresis of patients with prior SCT versus patients without prior SCT (*n* = 35). *p*‐values were calculated with Welch‐test. Occurrence of (F) CRS and (G) ICANS in patients receiving prior allo‐SCT (*n* = 27) versus in patients without receiving prior allo‐SCT (*n* = 106). *p*‐values were calculated using Chi‐square test.

In the HD‐CAR‐19 cohort, patients with ≤3 prior therapy lines had a trend toward higher T_N_‐cell fractions (*p* = .075; 31.81% ± 17.61% vs. 21.36% ± 10.41%) in the apheresis product and greater ex vivo fold expansion (*p* = .087; 27.12 ± 16.80 vs. 17.64 ± 8.90), compared to those with >3 therapy lines. They also had significantly fewer T_EM_‐cells (*p* = .036; 16.03% ± 11.67% vs. 25.79% ± 15.2%), and a trend toward lower T_CM_‐cell levels (*p* = .085; 12.27% ± 9.52% vs. 18.57% ± 11.58%) in their apheresis product. In the CAR T‐cell product, T_EFF_‐cells tended to be more abundant in the ≤3 therapy line group (*p* = .067; 29.49% ± 14.72% vs. 20.15% ± 11.45%) (Figure 4A–C).

Patients with prior allogeneic (allo) or autologous (auto) stem cell transplantation (SCT) showed a trend toward a lower CD4^+^/CD8^+^ ratio in the apheresis product (*p* = .057; 0.69 ± 0.51 vs. 1.11 ± 0.76) and significantly reduced fold expansion during manufacturing (*p* = .036; 17.19 ± 10.45 vs. 25.34 ± 13.55) (Figure 4D,E). Interestingly, prior allo‐SCT was linked to lower rates of CRS (*p* = .008) and ICANS (*p* = .015) following CAR T‐cell infusion (Figure 4F,G).

## 4 DISCUSSION

We analyzed 166 apheresis products from 154 patients with B‐cell malignancies to better understand parameters for optimal CD3^+^ T‐cell collection and the cellular composition relevant for CAR T‐cell therapy.

Across the cohort, lower CD3^+^ T‐cell yields were associated with markers of impaired hematopoiesis, such as reduced NC counts, lymphocyte counts, and hematocrit. In patients with NHL, an increasing number of prior therapy lines correlated with hematopoietic suppression, reflected in lower platelet and NC counts in the peripheral blood and apheresis product. This suggests that cumulative treatment exposure impairs hematopoiesis, which directly influences CD3^+^ T‐cell yield and the overall composition of apheresis products. Although CD3^+^ T‐cell yield in the apheresis product was not significantly associated with clinical response, pre‐apheresis peripheral blood lymphocyte count showed a significant difference between response groups. This suggests that overall immune fitness, rather than isolated CD3^+^ T‐cell quantity in the apheresis product, may be more relevant for therapeutic outcome, as noted before.^29^ Importantly, lymphocytes constitute a substantial fraction of the overall NC pool. Since NC counts decline with an increasing number of prior therapy lines, this represents an indirect yet clinically meaningful connection: heavily pretreated patients exhibit hematopoietic compromise, reducing lymphocyte availability, limiting CD3^+^ T‐cell yield and ultimately affecting CAR T‐cell product composition and therapeutic outcomes.

The lack of association between CD3^+^ T‐cell yield and response, along with a small effect size (*p* = .244, Cohen's *d* = 0.221) suggests that quantitative T‐cell abundance alone is insufficient to predict clinical efficacy. In contrast, pre‐apheresis lymphocyte count, which likely reflects broader biological dimensions—including cumulative treatment‐related immune damage, bone marrow reserve, and systemic immune competence—showed a statistically significant association with response and a moderate effect size (*p* = .044, Cohen's *d* = 0.378). Together, these findings support a biologically coherent model in which cumulative therapy exposure and overall immune fitness, rather than absolute T‐cell numbers, determine clinical outcome.^9^, ^29^, ^30^, ^31^

In 43 patients receiving apheresis for HD‐CAR‐19T‐cell production, both apheresis and final CAR T‐cell products were analyzed by immunophenotyping. Manufacturing led to an increase in T_CM_ and T_EM_ subsets, likely driven by the HD‐CAR‐19 construct, incorporating both CD28 and 4‐1BB co‐stimulatory domains favoring the expansion of different memory T‐cell subsets,^32^, ^33^ as well as Interleukin‐15 supplementation supporting T_CM_ development.^34^ The CD4^+^/CD8^+^ increased during manufacturing, reflecting the stronger ex vivo proliferative capacity of CD4^+^ T‐cells^15^, ^35^ and preferential expansion induced by the 4‐1BB domain.^33^ Patients with prior allo‐SCT had fewer CD4^+^ T‐cells in apheresis concentrates, possibly due to faster reconstitution of memory CD8^+^ T‐cells post‐transplantation.^36^, ^37^, ^38^ T‐cell proliferation during manufacturing positively correlated with naïve T‐cell abundance in the apheresis product—both proliferative activity and naïve T‐cell abundance are established indicators of T‐cell “fitness.”^30^, ^31^, ^39^

In patients with ALL treated with HD‐CAR‐19, CAR T‐cell product composition correlated with best response: patients achieving CR had higher proportions of T_EFF_, which mediate rapid immune responses. Across all patients in the HD‐CAR‐19 cohort, fewer prior therapy lines were associated with greater naïve T‐cell abundance and stronger proliferative capacity during manufacturing, resulting in a final product with higher effector T‐cell levels, as noted before.^9^, ^12^, ^39^, ^40^ This indicates that earlier apheresis preserves T‐cell fitness, producing a CAR T‐cell product with better expansion potential and composition conducive to effective immune responses—critical for optimal clinical outcomes, as illustrated in the ALL subgroup. While studies of CD19‐directed CAR T‐cell composition in ALL are limited, our findings differ from a CD22‐directed CAR T‐cell study, where best response after 28 days was linked to naïve and central memory subsets.^41^ Long‐term outcomes with CD19‐directed CAR T‐cells suggest that CD8^+^ naïve T‐cells promote survival,^42^ while Th2 deficiency is associated with relapse.^43^ In pediatric patients with B‐cell precursor ALL treated with tisa‐cel, treatment response was linked to natural killer and monocyte phenotypes in the peripheral blood rather than CAR T‐cell composition.^44^ In patients with LBCL reports vary: Lamure et al.^45^ reported that CD4^+^ and CD8^+^ T_EM_ cells were enriched in the CAR T‐cell product of patients with LBCL with best responses to axi‐cel and tisa‐cel. Other studies evaluated later time points: Locke et al.^39^ linked CD8^+^ CCR7^+^ CD45RA^+^ T‐cells in axi‐cel products to durable responses lasting at least two years. At three months, Deng et al.^10^ (axi‐cel) and Monfrini et al.^13^ (axi‐cel and tisa‐cel) identified T_CM_ cells in the infusion product as predictive of clinical benefit. Wang et al. further demonstrated in a phase 1 trial that CAR T‐cell products derived from T_CM_‐enriched aphereses lead to improved long‐term remissions.^46^ Rezvan et al.^47^ associated CD8^+^ T_EM_ cells in the CAR T‐cell product with responses at six months. Together, these studies suggest that different T‐cell subsets may drive initial versus sustained responses, depending on disease type and timing. T_EFF_ cells appear crucial for immediate responses, as in our ALL cohort, while memory subsets like T_EM_ and T_CM_ may support long‐term disease control, especially in LBCL.

In this study, axi‐cel, tisa‐cel, and brexu‐cel showed lower rates of CRS and ICANS than previously reported, with fewer severe events across all products.^6^, ^48^ However, direct comparisons are limited by differences in sample size, underlying disease, tumor burden, and prior treatments. Additionally, despite standardized criteria, variability in recognition, grading, and early management of CRS and ICANS may further contribute to differences in reported toxicity rates. As noted before,^49^, ^50^ HD‐CAR‐19 exhibited the most favorable safety profile, with 36% developing mostly grade 1/2 CRS and only one grade 1 ICANS. Patients with prior allo‐SCT experienced significantly lower rates of CRS and ICANS, consistent with earlier findings.^51^

These findings highlight the impact of prior therapies on T‐cell collection and apheresis and CAR T‐cell product composition. Heavily pretreated patients often display impaired hematopoiesis, resulting in lower CD3^+^ T‐cell yields and suboptimal product composition. Compromised product quality can influence outcomes, as higher T_EFF_ levels in the final product were linked to best responses in ALL. These observations support initiating apheresis earlier in the treatment course to preserve T‐cell fitness and generate CAR T‐cell products with optimal expansion potential and therapeutic efficacy.

## FUNDING INFORMATION

This work did not receive direct support in the form of grants, equipment, or pharmaceuticals.

## CONFLICT OF INTEREST STATEMENT

MS received funding for collaborative research from Apogenix, Hexal and Novartis, travel grants from Hexal and Kite, he received financial support for educational activities and conferences from bluebird bio, Kite and Novartis, he is a board member for MSD and (co‐)PI of clinical trials of MSD, GSK, Kite and BMS, as well as co‐Founder and shareholder of TolerogenixX Ltd. AS received travel grants from Hexal and Jazz Pharmaceuticals, research grant from Therakos/Mallinckrodt, consultant by Janssen‐Cilag and BMS and is co‐founder of TolerogenixX LtD. AS is part‐time employee of TolerogenixX Ltd. PDe received honorarium from MSD. KF received travel grants from Kite/Gilead and Pierre Fabre. M‐LS reports consultancy from Kite/Gilead, Takeda. PDr reports consultancy for AbbVie, AstraZeneca, Beigene, BMS, Gilead, Miltenyi (all to institution); speakers bureau for AbbVie, AstraZeneca, BeiGene, BMS, Gilead, Riemser, Roche (all to institution); research support from Riemser (all to institution); meeting attendance support from Beigene and Gilead; Participation on a Data Safety Monitoring Board for Novartis. The authors HK, FK, AH‐K, AL‐H, MSt declare that they have no COIs. All authors were asked to disclose potential COIs. The corresponding author received no disclosures from CM‐T, SS, TS, and BN, and therefore their COI status remains undetermined.

## Supporting information


**Supplementary Figure 1.** Gating strategy for T‐cell immunophenotypes.

**Supplementary Figure 2.** Correlation of pre‐apheresis parameters and CD3^+^ T‐cell yield. Assessment of CD3^+^ T‐cell yield by pre‐apheresis NC count (*n* = 165) (A), hct (*n* = 165) (B), and hb (*n* = 165) (C). *P*‐ and *R*‐values were calculated using Spearman's correlation.

**Supplementary Figure 3.** Proportion of T‐cell immunophenotypes in apheresis and product comparing different types of disease. Kruskal–Wallis test and post hoc tests with Bonferroni correction were used to compare T‐cell subsets among disease subgroups with a sufficient sample size (*n* >5) in apheresis (A) and product (B).

**Supplementary Figure 4.** Correlation of T‐cell immunophenotypes in the apheresis and product and fold expansion during CAR T‐cell production. Analysis of fold expansion by proportion of (A) T_N_ (*n* = 42), (B) T_CM_ (*n* = 42), (C) T_EM_ (*n* = 42), (D) T_EFF_ (*n* = 42) in the apheresis. Evaluation of (E)T_N_ (*n* = 42), (F) T_CM_ (*n* = 42), (G) T_EM_ (*n* = 42), (H) T_EFF_ (*n* = 42) in the product by fold expansion. (I) Analysis of fold expansion by CD4^+^/CD8^+^ ratio in apheresis (*n* = 40). Evaluation of CD4^+^/CD8^+^ ratio in product by fold expansion (*n* = 42). *R*‐ and *P*‐values were calculated using Spearman's correlation.

**Supplementary Figure 5.** T‐cell immunophenotypes and CD4^+^/CD8^+^ ratio in patients with CLL in the HD‐CAR‐19 cohort. (A, B) Analysis of T‐cell immunophenotypes in apheresis and product of patients with CLL (*n* = 8) by response (CR vs. non‐CR). *P*‐values were calculated using student's *t*‐test. (C, D) Evaluation of CD4^+^/CD8^+^ ratio in apheresis and product of patients with CLL (*n* = 8) by response. *P*‐values were calculated using student's *t*‐test.

**Supplementary Figure 6.** Correlation between the number of prior therapy lines and cell counts in both peripheral blood and apheresis product of patients with NHL. Analysis of (A) NC count (*n* = 134), (B) platelet count (*n* = 133), (C) granulocytes (*n* = 134), and (D) monocytes (*n* = 134) in the peripheral blood pre‐apheresis by number of prior therapy lines. Assessment of (E) NC (*n* = 134) and (F) platelet count (*n* = 120) in the apheresis by number of previous therapy lines. *R*‐ and *P*‐values were calculated using Spearman's correlation. Results are shown as mean ± SD.

**Supplementary Table 1.** Requirements for CAR T‐cell products.
**Supplementary Table 2**. Patients' characteristics in the HD‐CAR‐19 cohort.
**Supplementary Table 3**. Reasons for second apheresis.
**Supplementary Table 4**. Deaths Following CAR T‐cell Therapy.

## Data Availability

The data that support the findings of this study are available from the corresponding author upon reasonable request.
