# Supplementary figures and images for: Impaired hematopoiesis affects apheresis and CAR T‐cell product composition and treatment response
Source: Transfusion. 2026 Apr 10;66(7):1375–89. doi: 10.1111/trf.70224 (PMC13350226; doi:10.1111/trf.70224)

Suppl. Figure 1

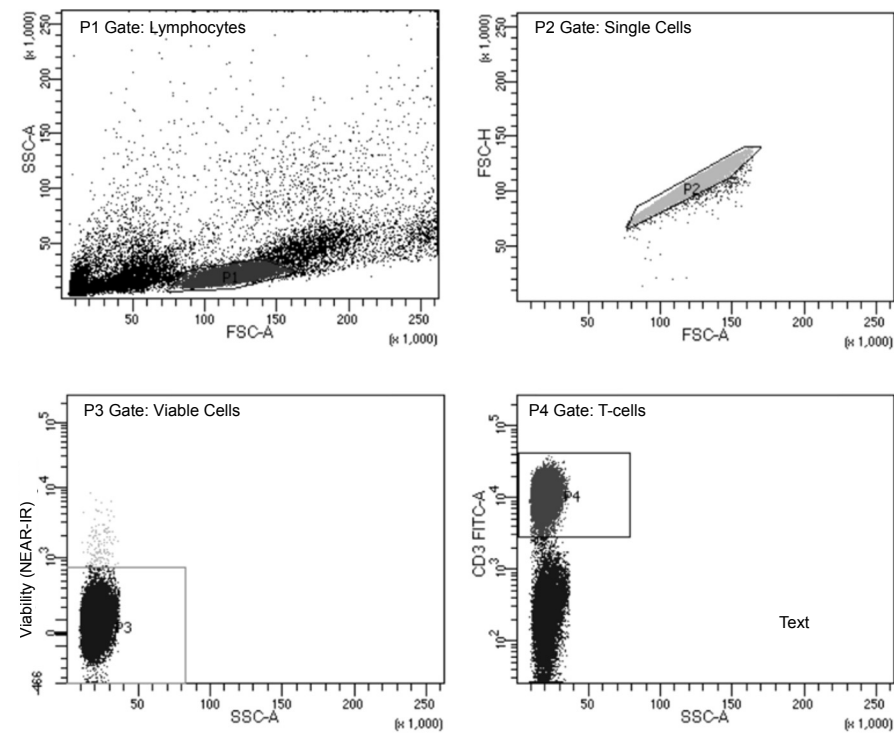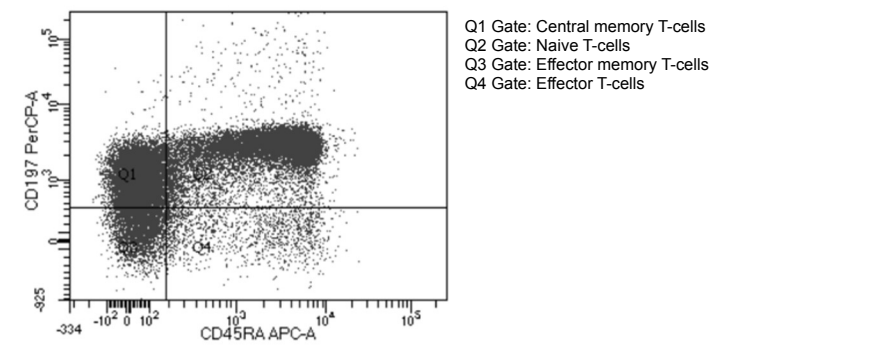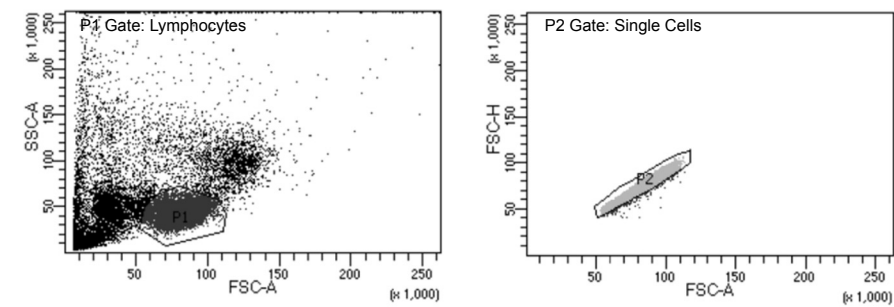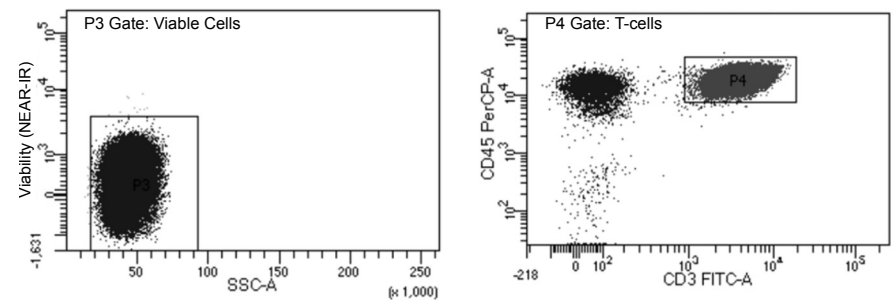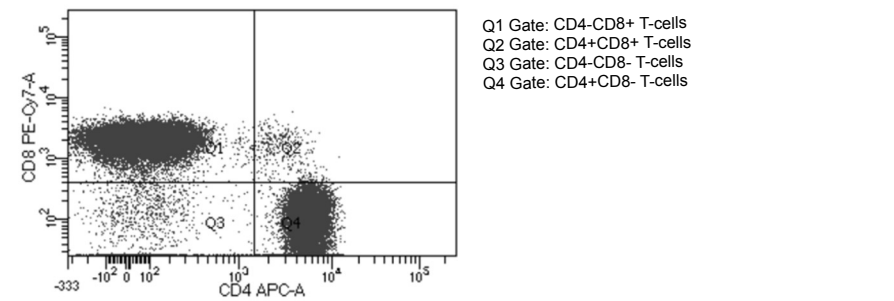

Supplement: Supplementary file 1 — Supplementary Figure 1. Gating strategy for T‐cell immunophenotypes. [file TRF-66-1375-s004.pdf]

**Suppl. Figure 2A**

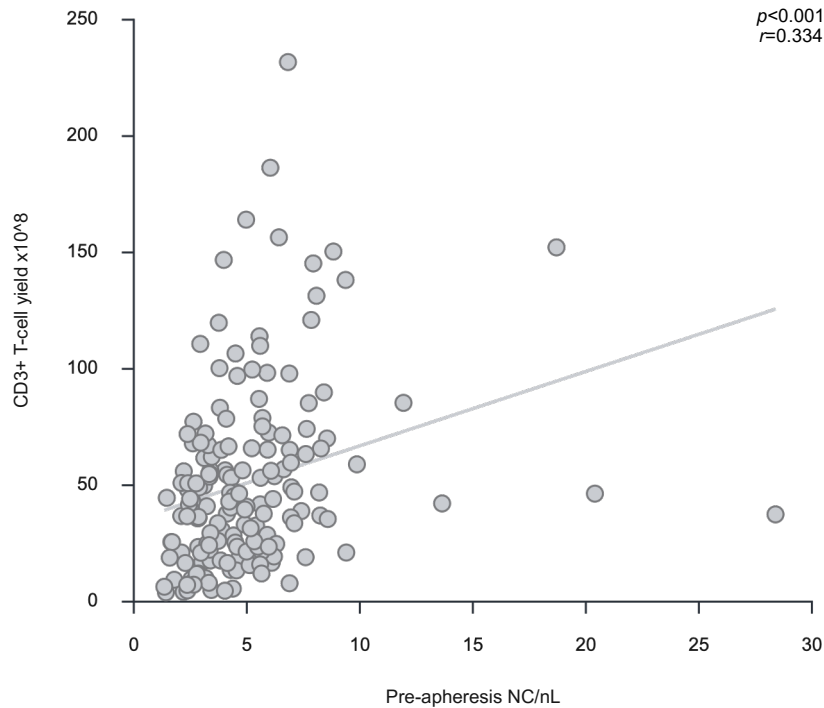

**Suppl. Figure 2B**

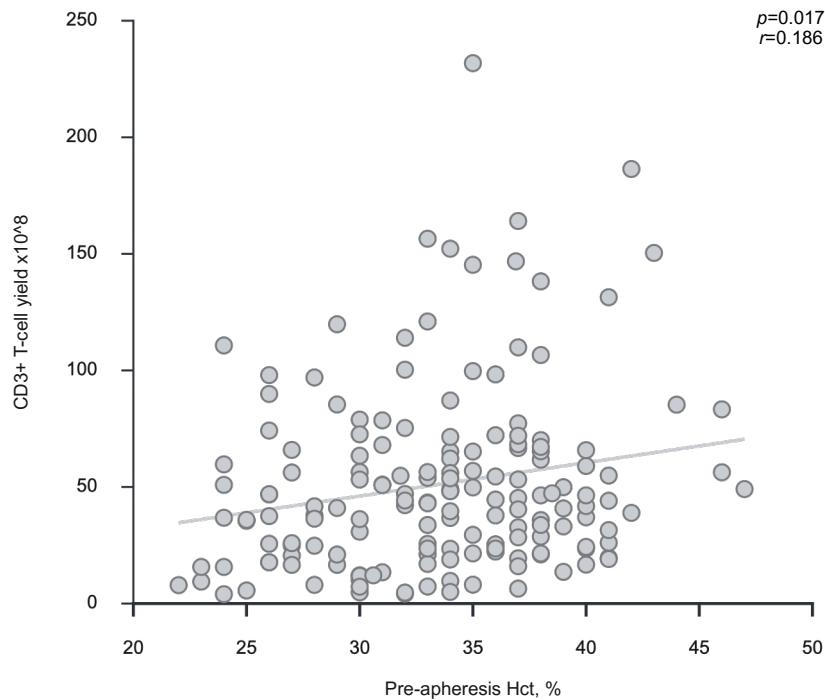

Suppl. Figure 2C

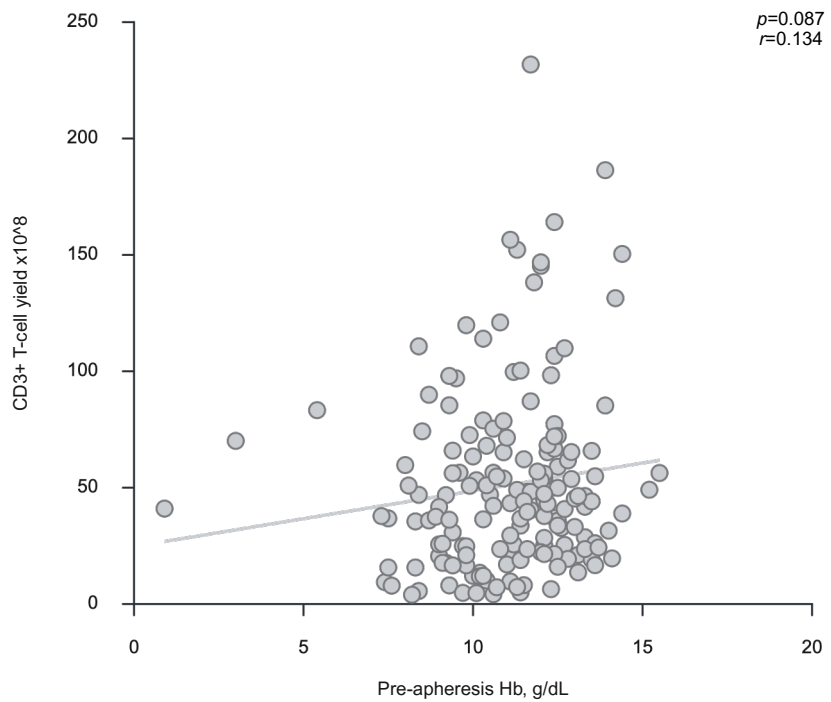

Supplement: Supplementary file 2 — Supplementary Figure 2. Correlation of pre‐apheresis parameters and CD3+ T‐cell yield. Assessment of CD3+ T‐cell yield by pre‐apheresis NC count (n = 165) (A), hct (n = 165) (B), and hb (n = 165) (C). P‐ and R‐values were calculated using Spearman's correlation. [file TRF-66-1375-s007.pdf]

**Suppl. Figure 5A**

CLL (n=8)

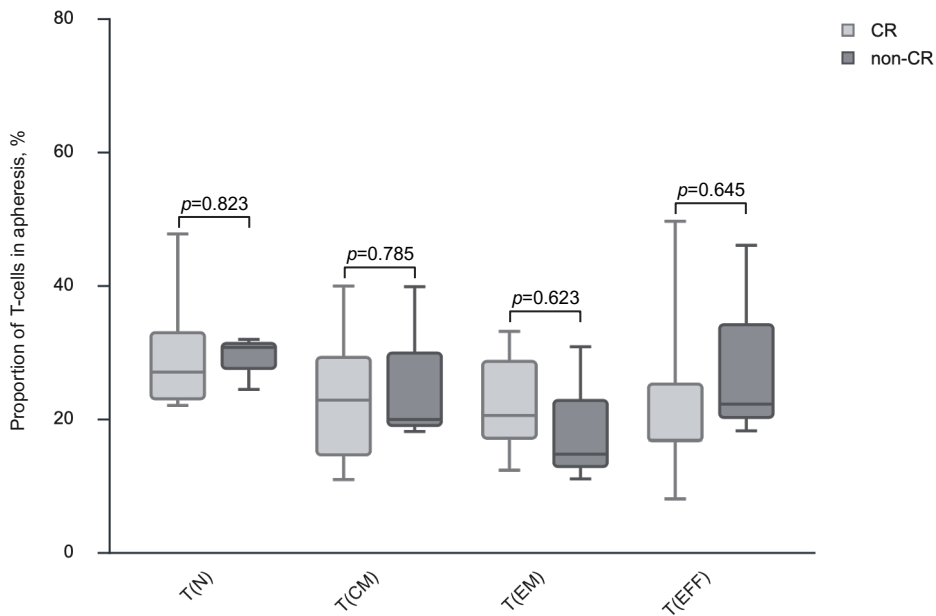

**Suppl. Figure 5B**

CLL (n=8)

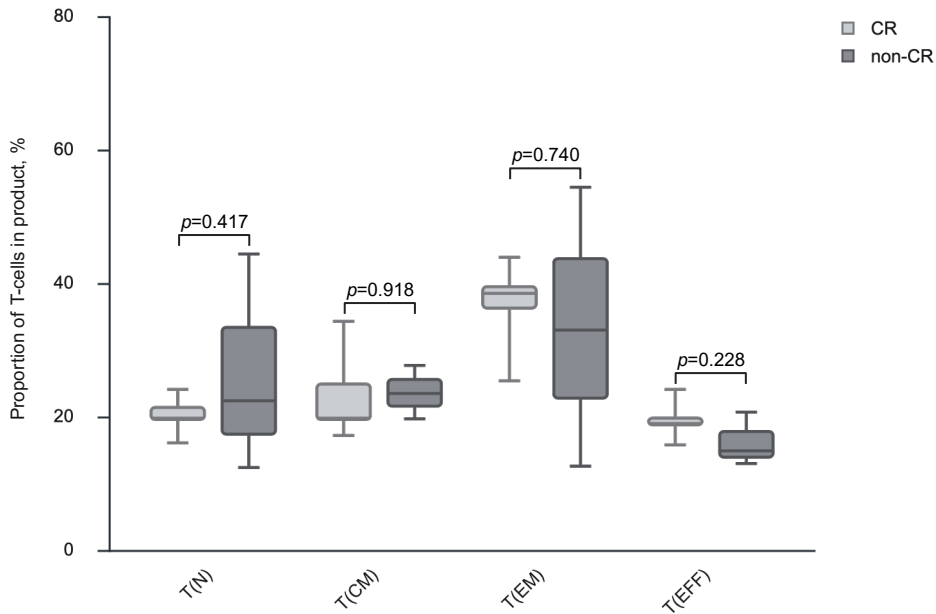

**Suppl. Figure 5C**

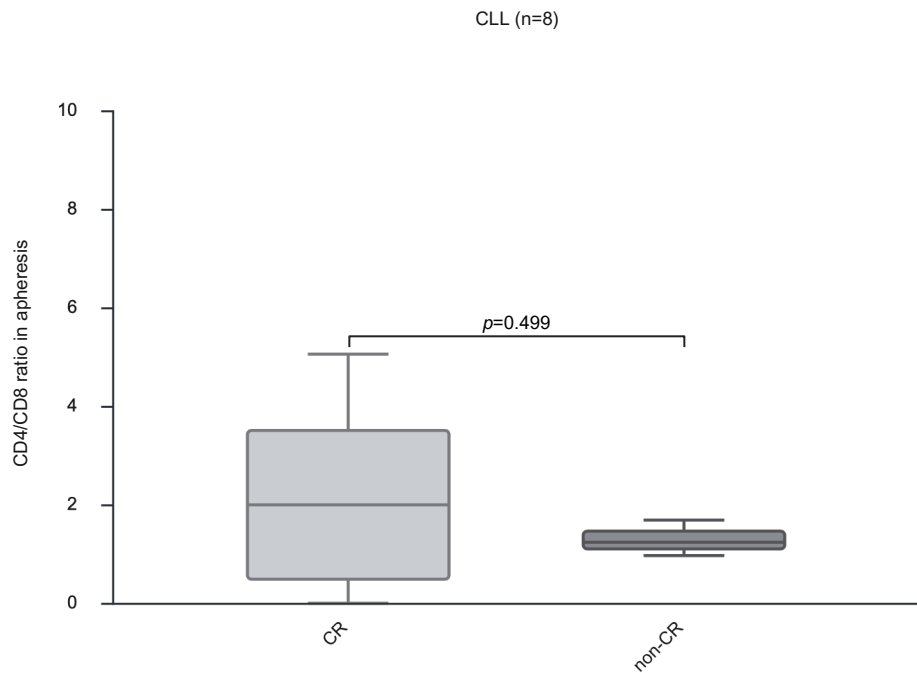

**Suppl. Figure 5D**

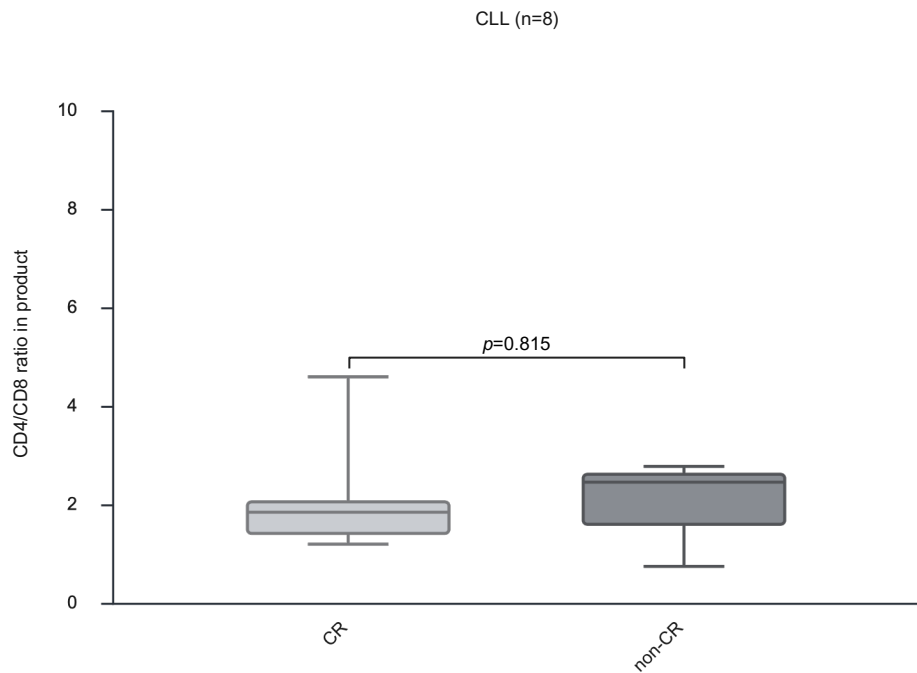

Supplement: Supplementary file 5 — Supplementary Figure 5. T‐cell immunophenotypes and CD4+/CD8+ ratio in patients with CLL in the HD‐CAR‐19 cohort. (A, B) Analysis of T‐cell immunophenotypes in apheresis and product of patients with CLL (n = 8) by response (CR vs. non‐CR). P‐values were calculated using student's t‐test. (C, D) Evaluation of CD4+/CD8+ ratio in apheresis and product of patients with CLL (n = 8) by response. P‐values were calculated using student's t‐test. [file TRF-66-1375-s006.pdf]

Suppl. Figure 6A

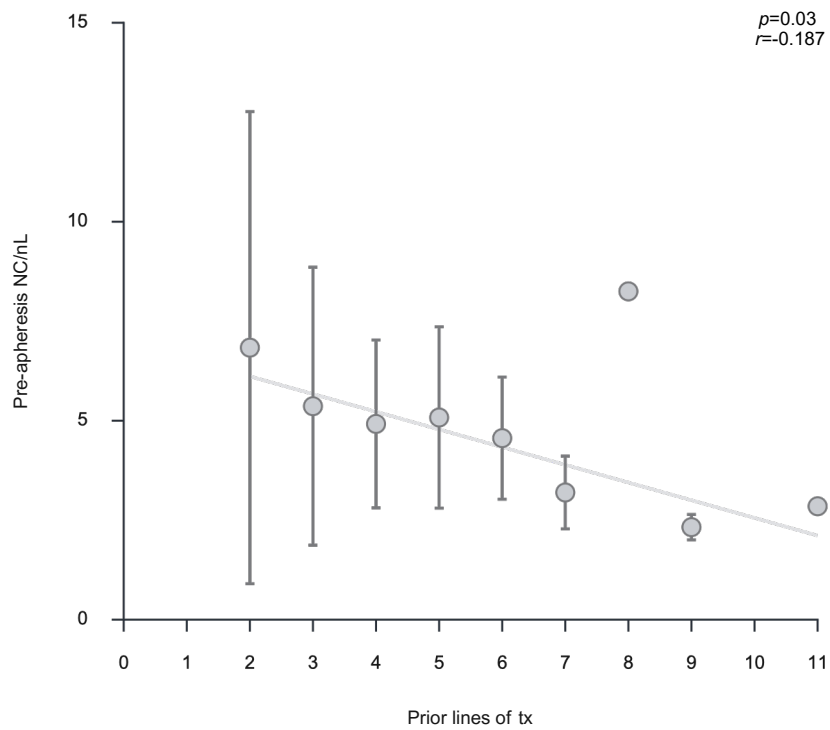

**Suppl. Figure 6B**

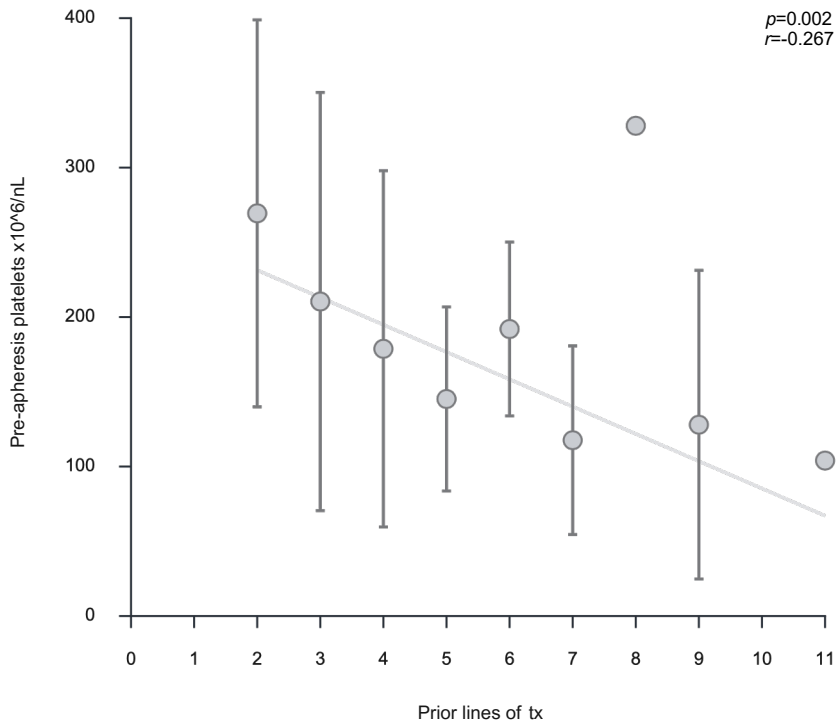

**Suppl. Figure 6C**

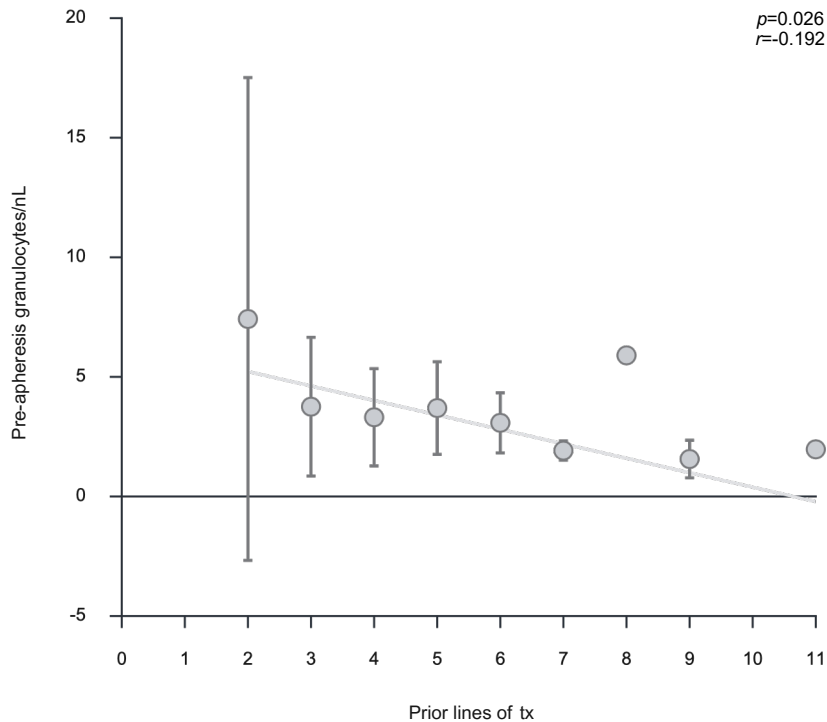

Suppl. Figure 6D

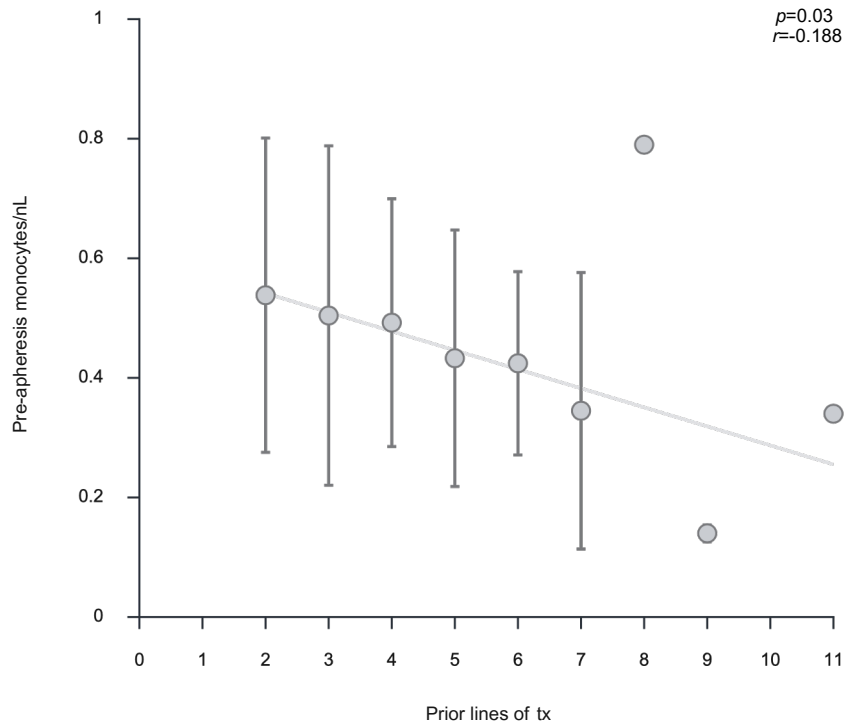

Suppl. Figure 6E

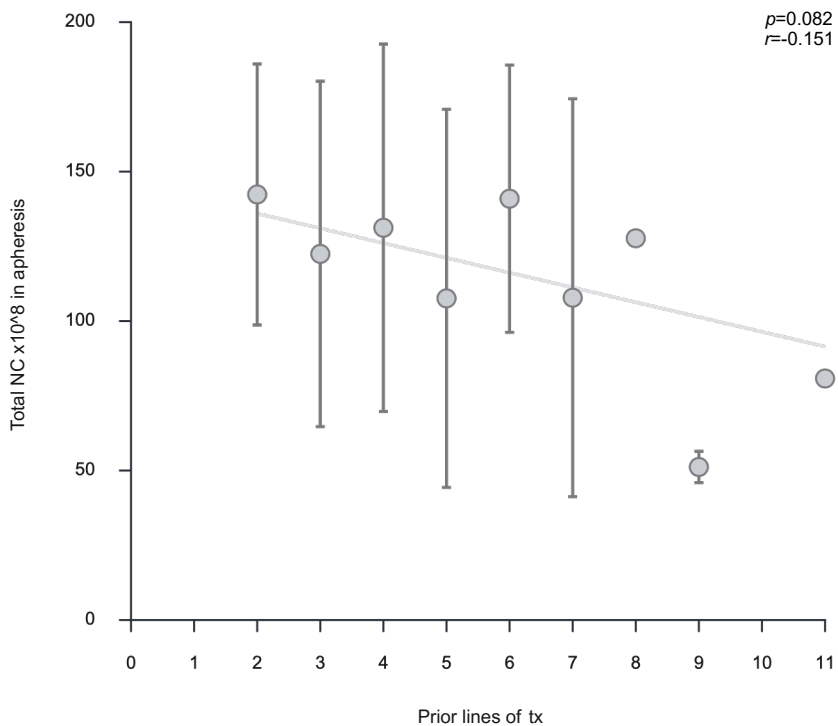

Suppl. Figure 6F

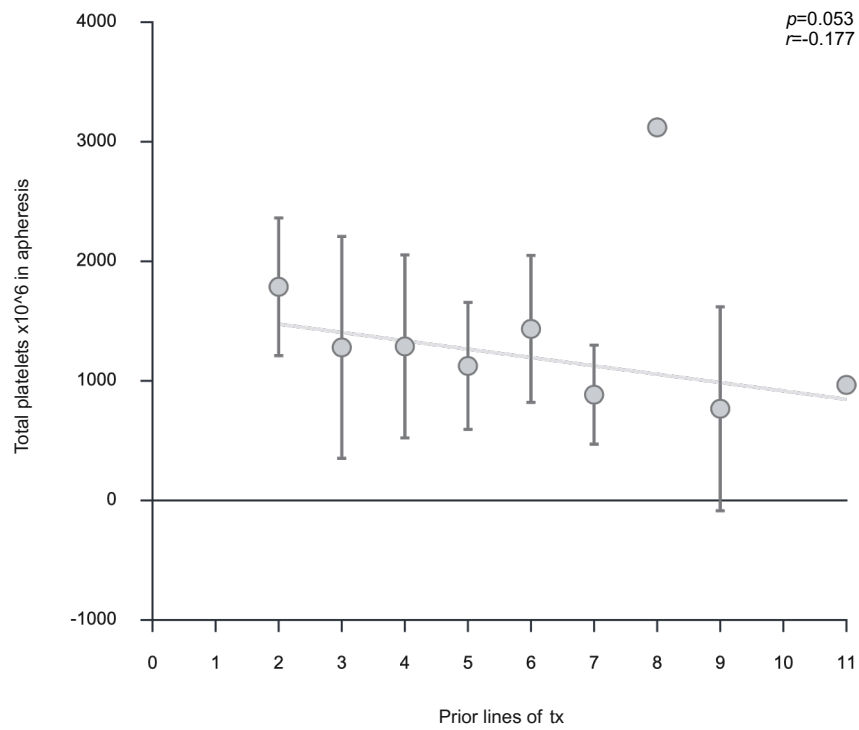

Supplement: Supplementary file 6 — Supplementary Figure 6. Correlation between the number of prior therapy lines and cell counts in both peripheral blood and apheresis product of patients with NHL. Analysis of (A) NC count (n = 134), (B) platelet count (n = 133), (C) granulocytes (n = 134), and (D) monocytes (n = 134) in the peripheral blood pre‐apheresis by number of prior therapy lines. Assessment of (E) NC (n = 134) and (F) platelet count (n = 120) in the apheresis by number of previous therapy lines. R‐ and P‐values were calculated using Spearman's correlation. Results are shown as mean ± SD. [file TRF-66-1375-s005.pdf]
