# Supplementary material for: Impaired hematopoiesis affects apheresis and CAR T‐cell product composition and treatment response
Source: Transfusion. 2026 Apr 10;66(7):1375–89. doi: 10.1111/trf.70224 (PMC13350226; doi:10.1111/trf.70224)
Supplement: Supplementary file 3 — Supplementary Figure 3. Proportion of T‐cell immunophenotypes in apheresis and product comparing different types of disease. Kruskal–Wallis test and post hoc tests with Bonferroni correction were used to compare T‐cell subsets among disease subgroups with a sufficient sample size (n >5) in apheresis (A) and product (B). [file TRF-66-1375-s001.pdf]

Suppl. Figure 3A

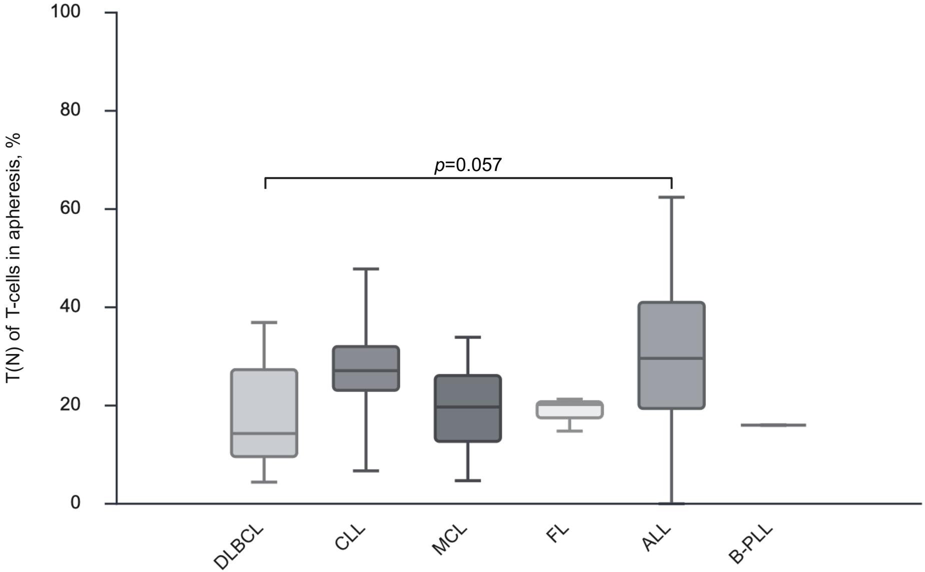

Kruskal–Wallis test: p=0.259. Bonferroni-adjusted post-hoc p-values are displayed in the figure only when <1.0.

Suppl. Figure 3B

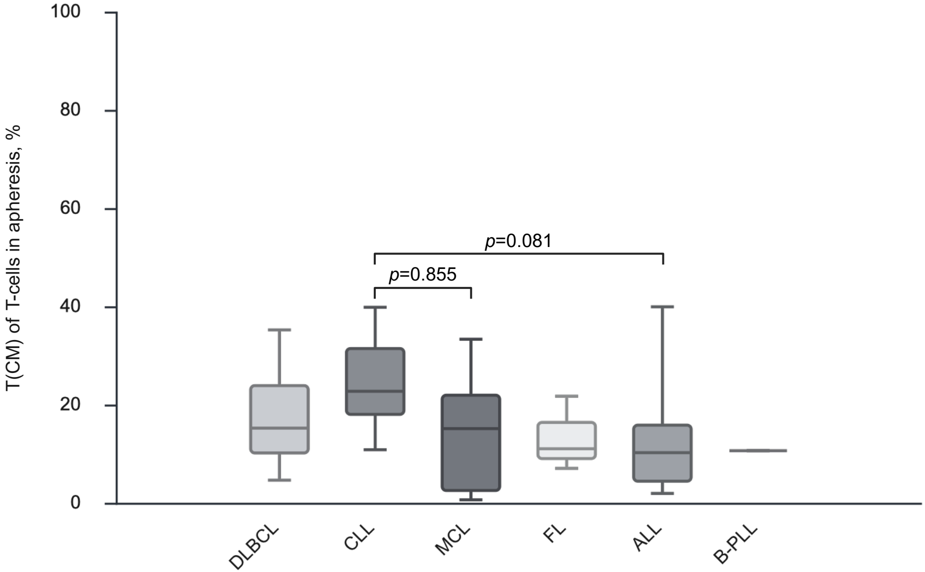

Kruskal–Wallis test: *p*=0.111. Bonferroni-adjusted post-hoc p-values are displayed in the figure only when <1.0.

Suppl. Figure 3C

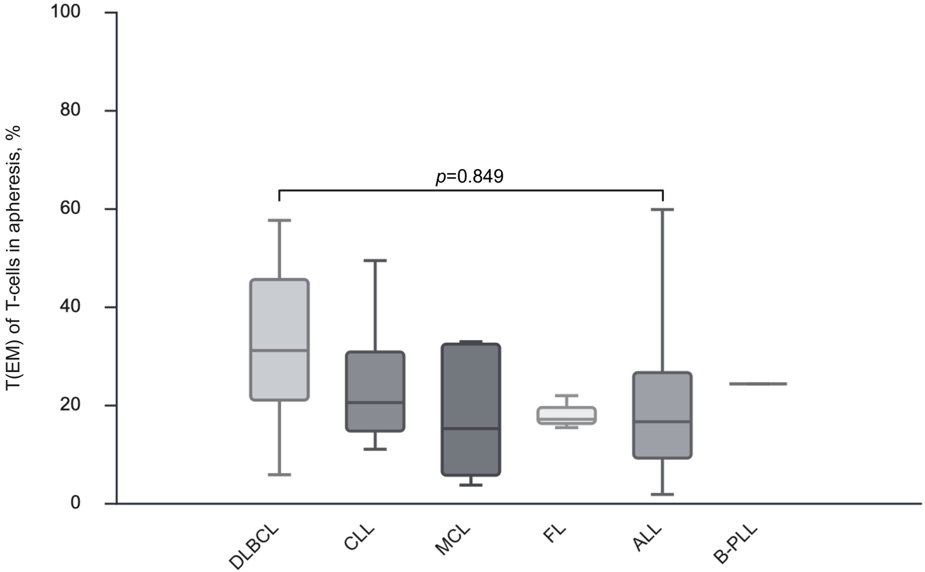

Kruskal–Wallis test: *p*=0.408. Bonferroni-adjusted post-hoc p-values are displayed in the figure only when <1.0.

Suppl. Figure 3D

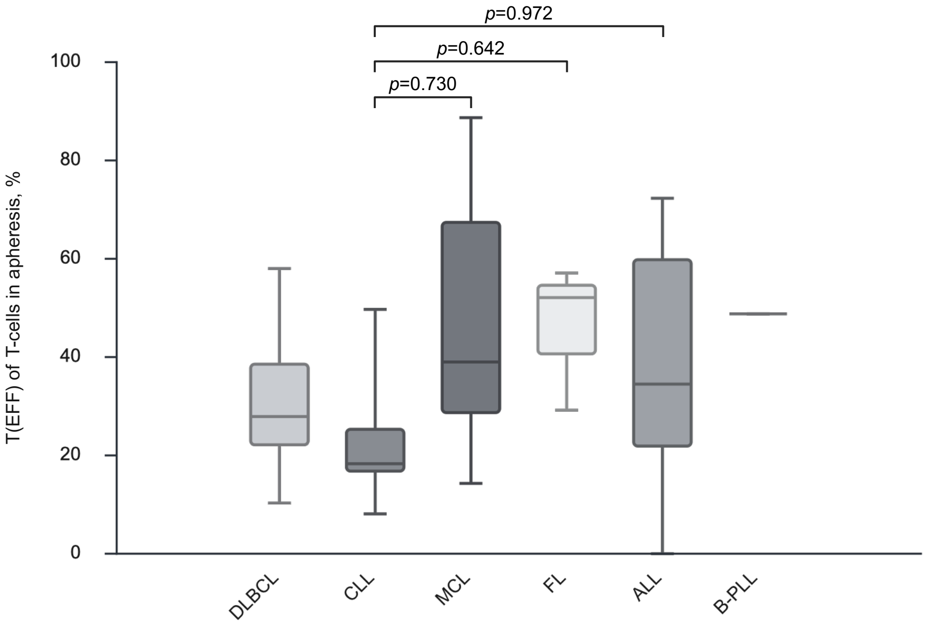

Kruskal–Wallis test: *p*=0.234. Bonferroni-adjusted post-hoc p-values are displayed in the figure only when <1.0.

Suppl. Figure 3E

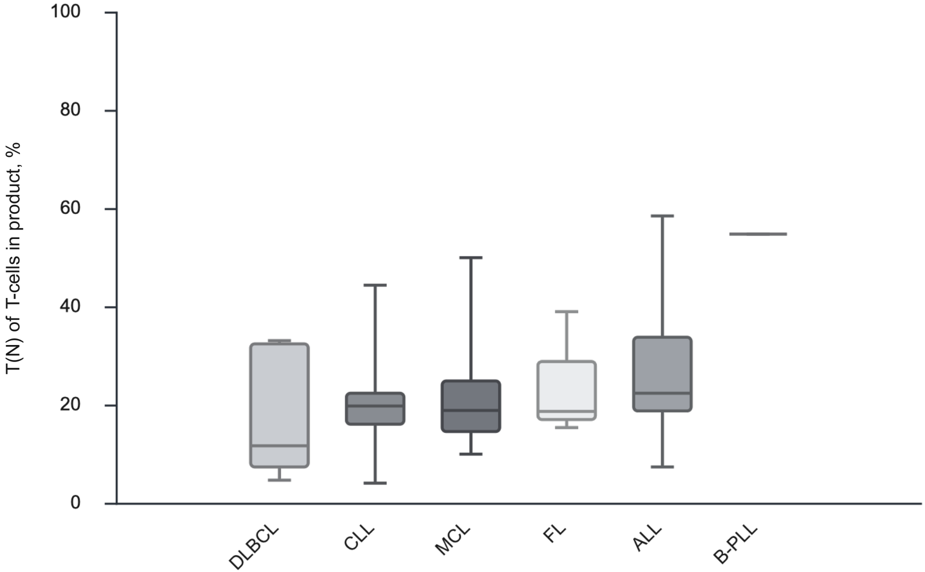

Kruskal–Wallis test: *p*=0.637. All Bonferroni-adjusted post-hoc p-values were 1.0 and are therefore not displayed.

Suppl. Figure 3F

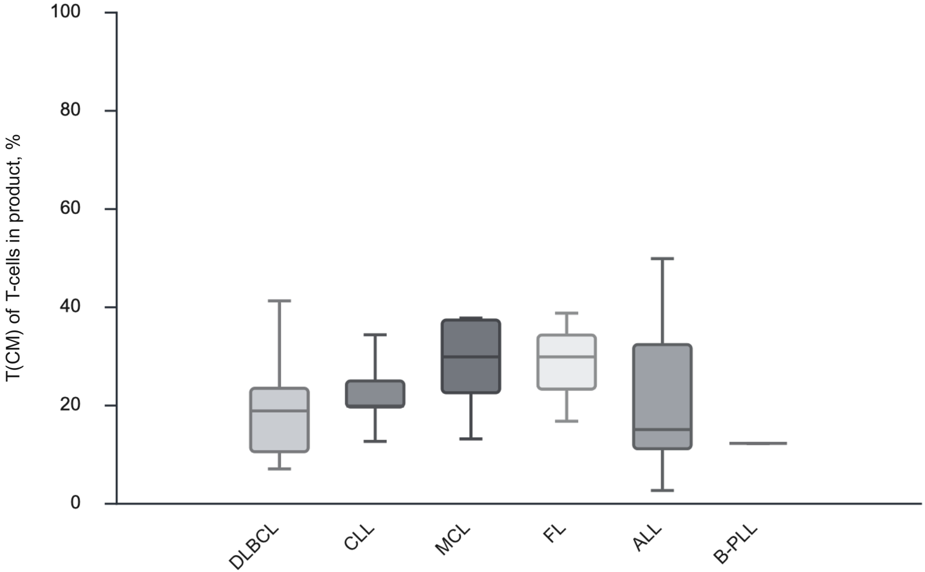

Kruskal–Wallis test: *p*=0.417. All Bonferroni-adjusted post-hoc p-values were 1.0 and are therefore not displayed.

Suppl. Figure 3G

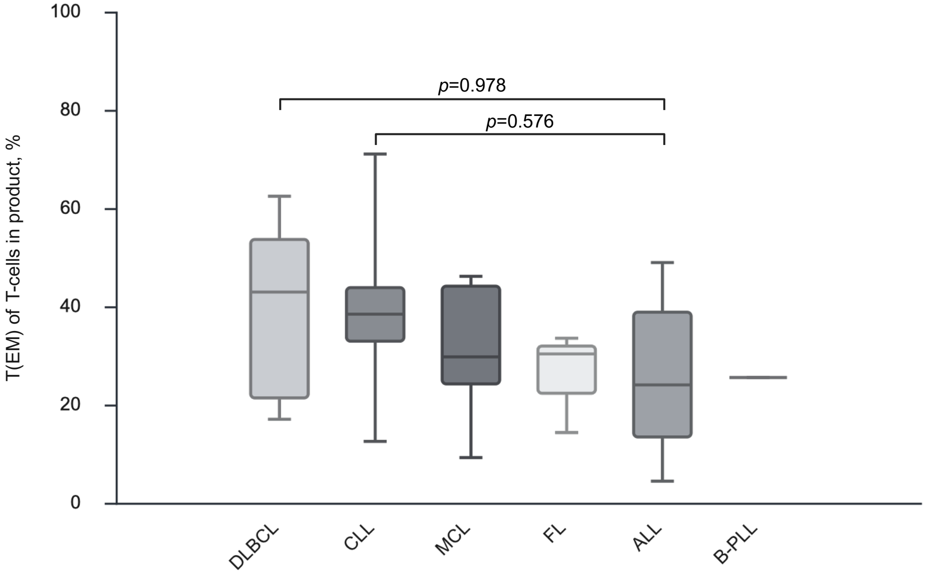

Kruskal–Wallis test: p=0.283. Bonferroni-adjusted post-hoc p-values are displayed in the figure only when <1.0.

Suppl. Figure 3H

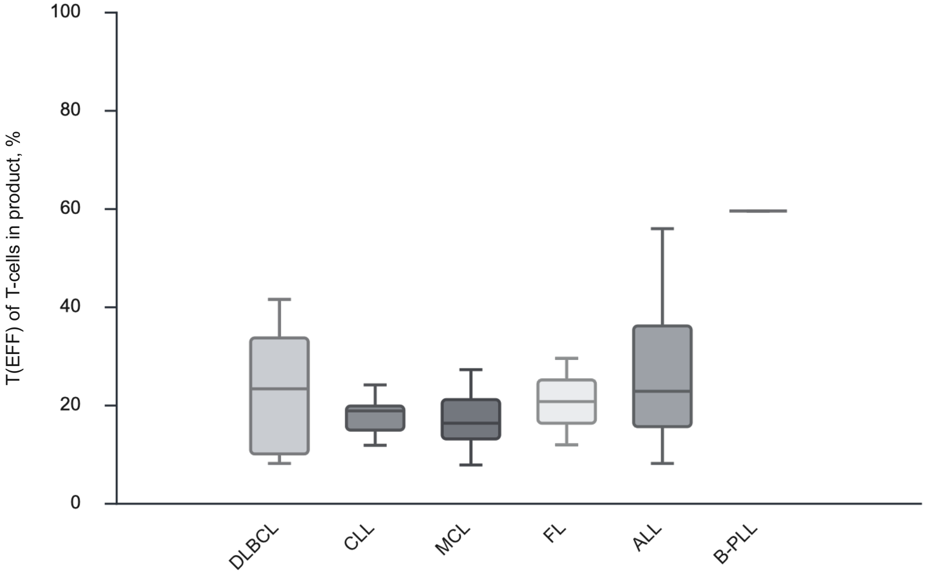

Kruskal–Wallis test: *p*=0.600. All Bonferroni-adjusted post-hoc p-values were 1.0 and are therefore not displayed.
