# Supplementary material for: Impaired hematopoiesis affects apheresis and CAR T‐cell product composition and treatment response
Source: Transfusion. 2026 Apr 10;66(7):1375–89. doi: 10.1111/trf.70224 (PMC13350226; doi:10.1111/trf.70224)

Suppl. Figure 4A

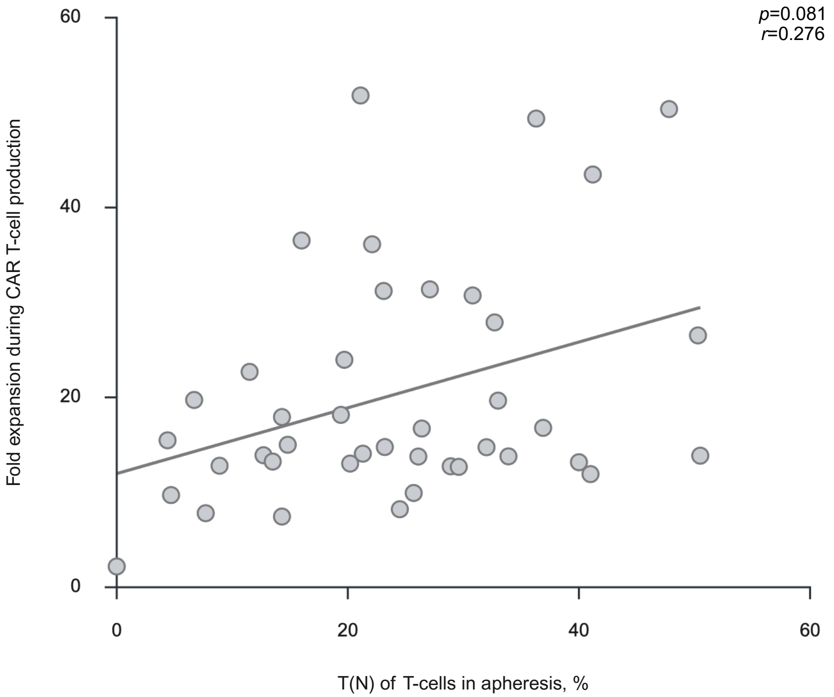

Suppl. Figure 4B

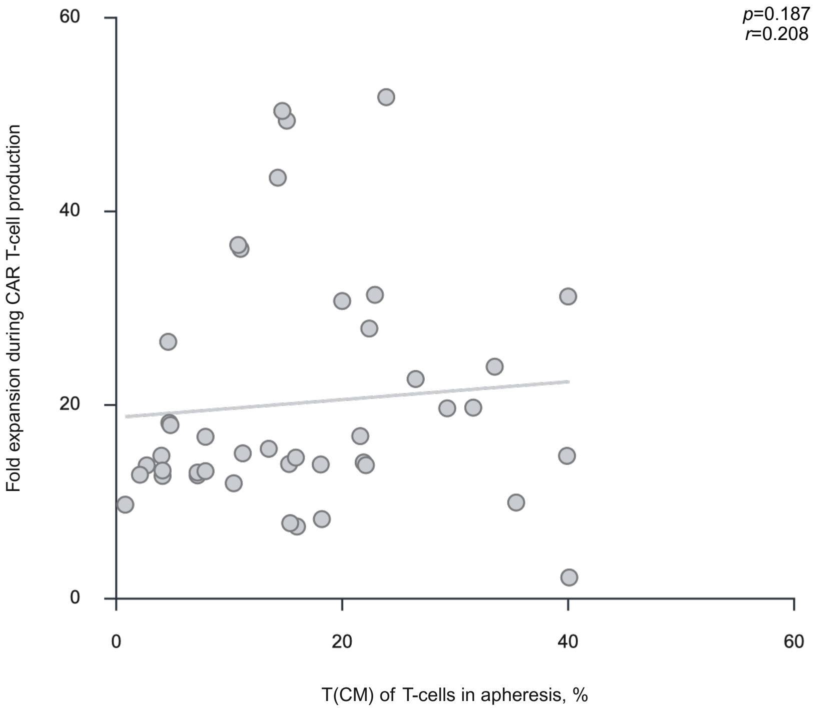

Suppl. Figure 4C

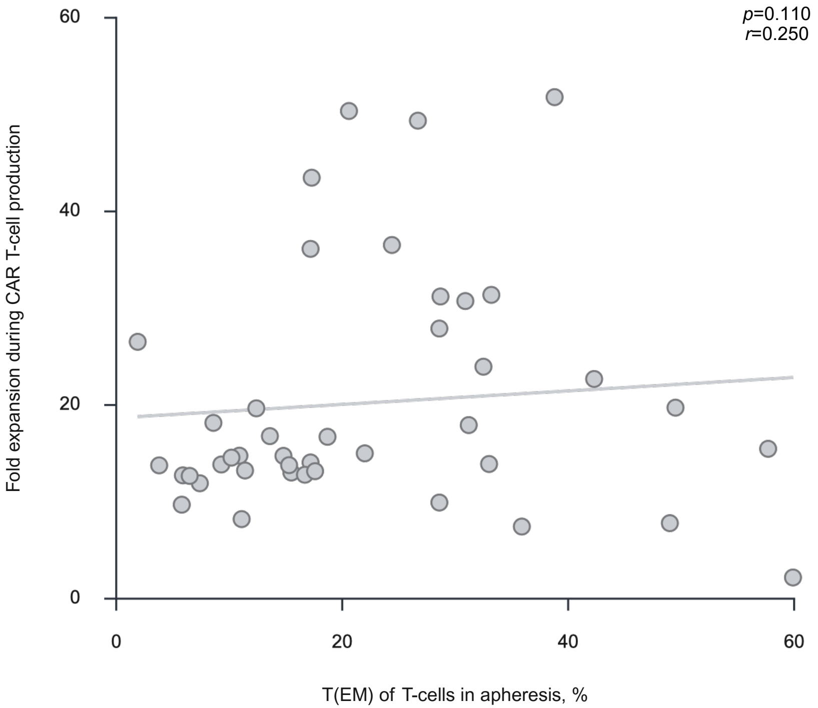

Suppl. Figure 4D

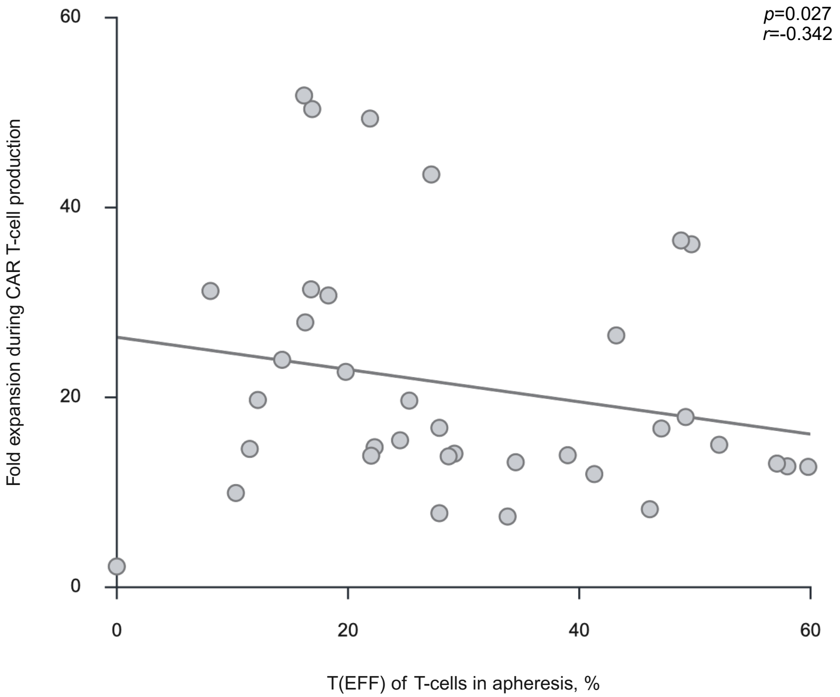

Suppl. Figure 4E

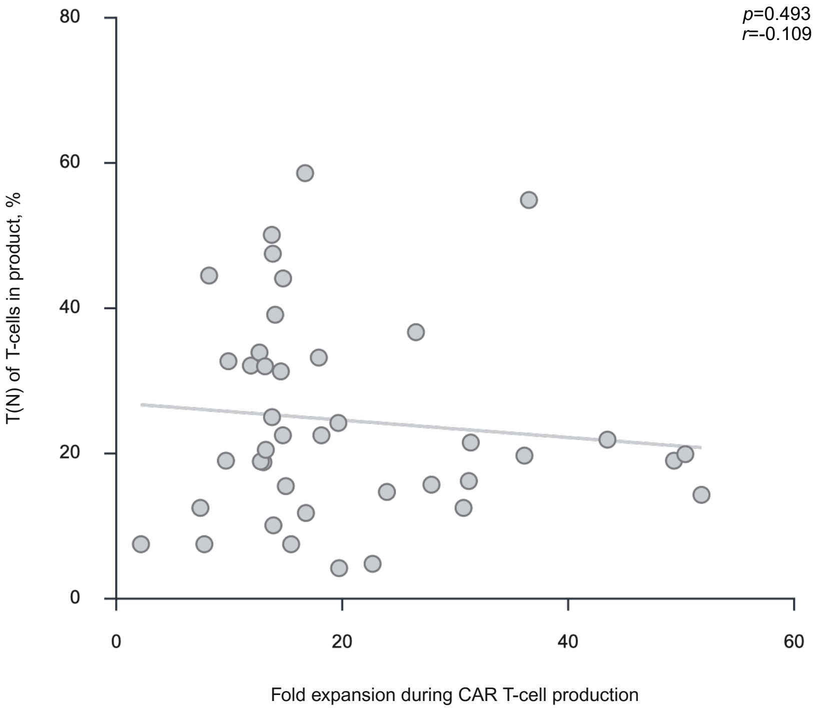

Suppl. Figure 4F

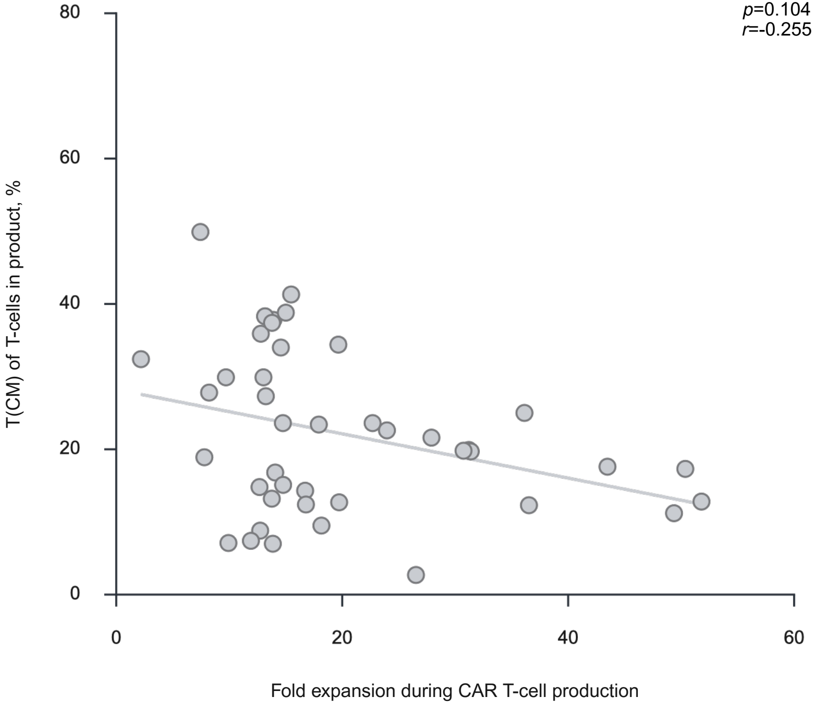

Suppl. Figure 4G

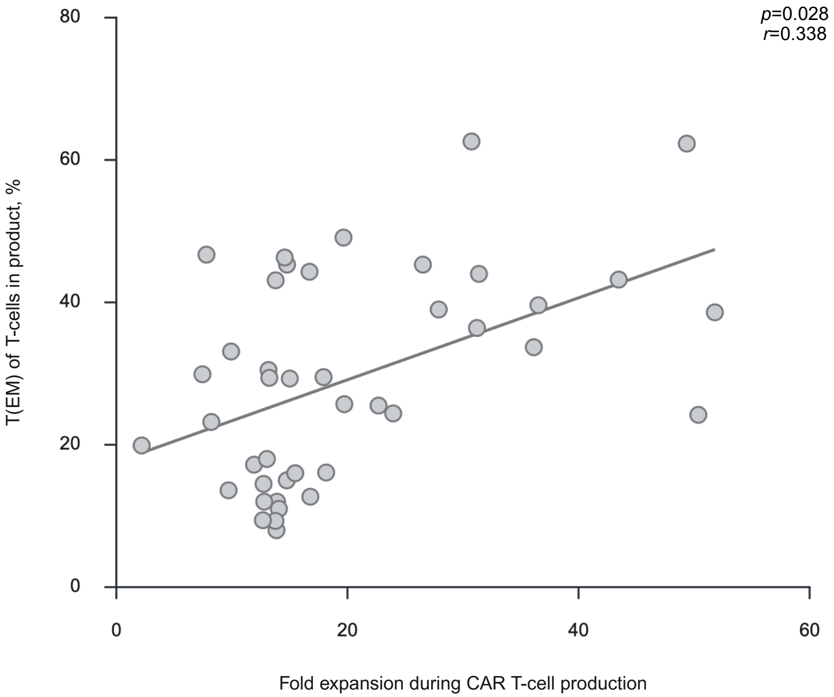

Suppl. Figure 4H

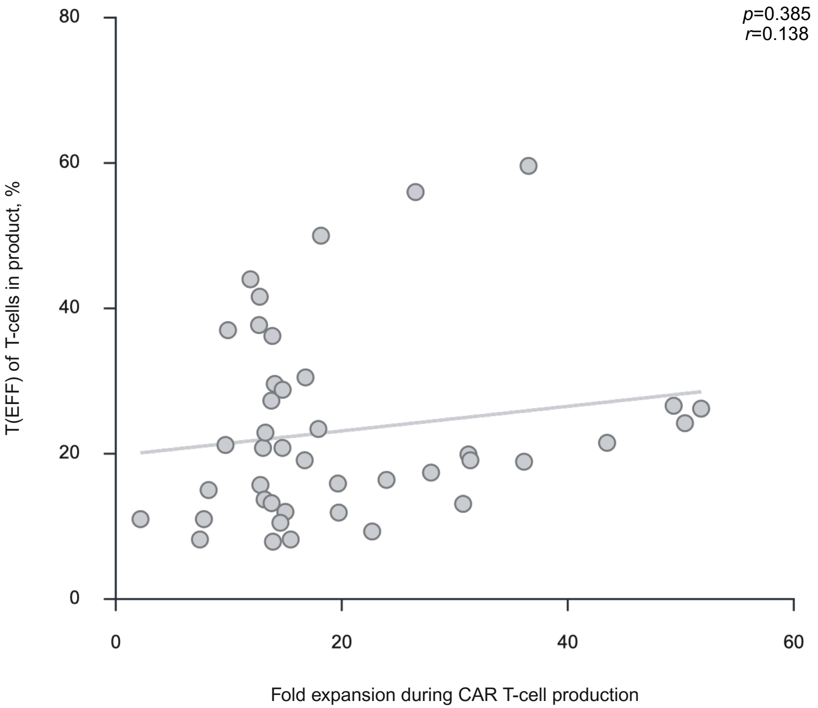

Suppl. Figure 4I

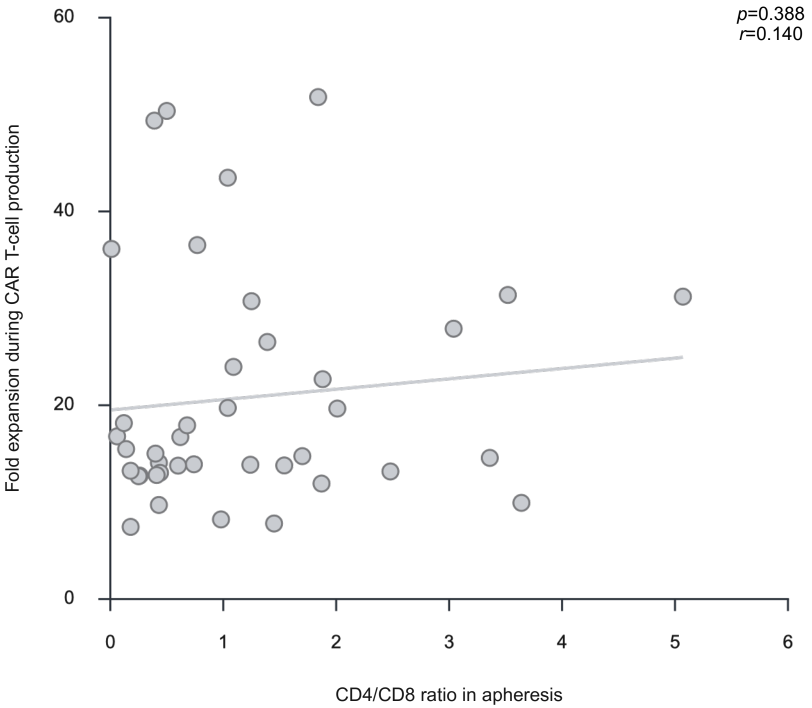

Suppl. Figure 4J

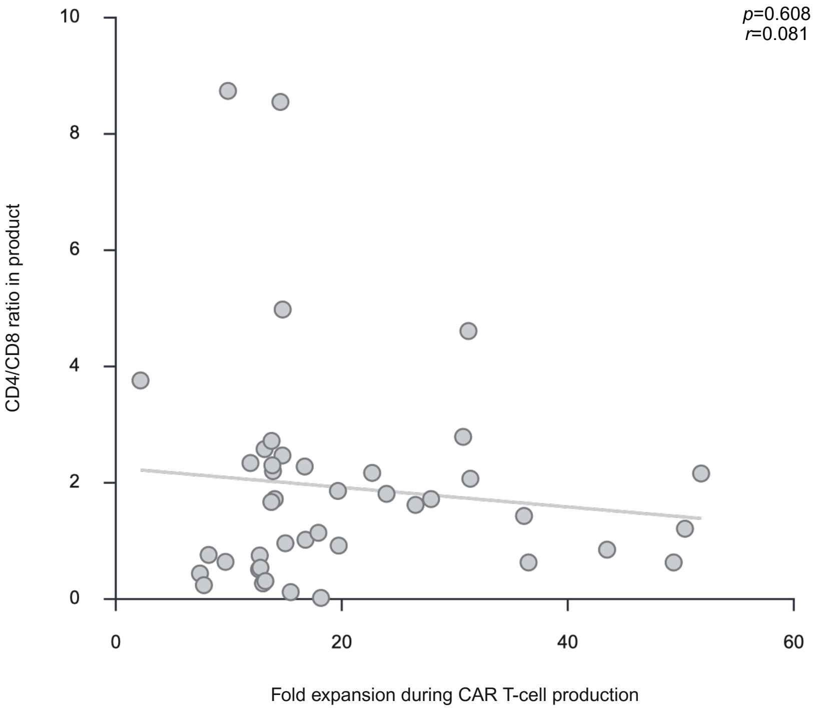

Supplement: Supplementary file 4 — Supplementary Figure 4. Correlation of T‐cell immunophenotypes in the apheresis and product and fold expansion during CAR T‐cell production. Analysis of fold expansion by proportion of (A) TN (n = 42), (B) TCM (n = 42), (C) TEM (n = 42), (D) TEFF (n = 42) in the apheresis. Evaluation of (E)TN (n = 42), (F) TCM (n = 42), (G) TEM (n = 42), (H) TEFF (n = 42) in the product by fold expansion. (I) Analysis of fold expansion by CD4+/CD8+ ratio in apheresis (n = 40). Evaluation of CD4+/CD8+ ratio in product by fold expansion (n = 42). R‐ and P‐values were calculated using Spearman's correlation. [file TRF-66-1375-s002.pdf]
