# Supplementary material for: Impaired hematopoiesis affects apheresis and CAR T‐cell product composition and treatment response
Source: Transfusion. 2026 Apr 10;66(7):1375–89. doi: 10.1111/trf.70224 (PMC13350226; doi:10.1111/trf.70224)
Supplement: Supplementary file 7 — Supplementary Table 1. Requirements for CAR T‐cell products. Supplementary Table 2. Patients' characteristics in the HD‐CAR‐19 cohort. Supplementary Table 3. Reasons for second apheresis. Supplementary Table 4. Deaths Following CAR T‐cell Therapy. [file TRF-66-1375-s003.pdf]

Suppl. Tables

Suppl. Table 1. Requirements for CAR T-cell products

CAR-T cell product	Indication	Collection target	Lymphodepleting chemotherapy, mg/m^2	CAR T-cell dose	Time thawing - transfusion
**Axi-cel**	r/r DLBCL, PMBCL, FL	5 - 10 x 10^9 TNCs	C 500, F 30 on d-5, d-4, d-3	1 - 2 x 10^6/kg	3 h
**Tisa-cel**	r/r B-ALL ≤25 y/o, DLBCL, FL	≥ 2 x 10^9 TNCs and<br>≥ 1 x 10^9 CD3$^+$ and<br>≥ 3 % CD3$^+$	B-ALL: C 500/d for 2d, F 30/d for 4d between d-6 and d-2<br>DLBCL, FL: C 250/d for 3d, F 25/d for 3d between d-6 and d-2	ALL: patients < 50 kg 0.2 - 5 x 10^6/kg patients > 50 kg 0.1 - 2.5 x 10^8<br>DLBCL, FL: 0.6 - 6 x 10^8	30 min
**Brexu-cel**	r/r MCL, B-ALL >26y/o	5 - 10 x 10^9 TNCs	MCL: C 500, F 30 on d-5, d-4, d-3<br>ALL: C 900 on d-2, F 25 on d-4, d-3 or d2	MCL: 1 x 10^6 - 2 x 10^6/kg<br>ALL: 1 x 10^6/kg	30 min
**Liso-cel**	r/r DLBCL, PMBCL, FL3B	Processed volume: 12 L<br>(< 1/nL lymphocytes)	C 300/d for 3d, F 30/d for 3d between d-7 and d-2	44 - 120 x 10^6	2 h
**HD-CAR-19**	r/r B-ALL, CLL, DLBCL, FL, MCL	≥ 2 x 10^9 TNCs and<br>≥ 1 x 10^9 CD3$^+$	C 500/d for 3d, F 30/d for 3 d between d-5 and d-2	Dose I: 1 x 10^6/m^2<br>Dose II: 5 x 10^6/ m^2<br>Dose III: 20 x 10^6/ m^2<br>Dose IV: 50 x 10^6/ m^2<br>Dose V: 100 x 10^6/ m^2<br>Dose VI 200 x 10^6/ m^2	2 h

Abbreviations: ALL, acute lymphocytic leukemia; C, cyclophosphamide; CLL, chronic lymphocytic leukemia; DLBCL, diffuse large B-cell lymphoma; F, fludarabine; FL, follicular lymphoma; FL3B, follicular lymphoma grade 3B; MCL, mantle cell lymphoma; ped, pediatric; PMBCL, primary mediastinal B-cell lymphoma; r/r, relapsed or refractory; TNC, total nucleated cells; y/o, years old

Suppl. Table 2. Patients' characteristics in the HD-CAR-19 cohort

Disease	CAR T-cell Dose, x 10^6/m2 BSA	Patient Data					Peripheral Blood prior to Apheresis			Apheresis			Best Response
		Gender	Age	Prior therapy lines	Prior allo-SCT	Prior auto-SCT	WBC/nL	Lymphocytes /nL	Lymphocytes/WBC, %	TNC, x10^8	$CD3^+$ T-cells, x10^8	$CD3^+$ /TNC, %	
ALL	1	m	21	-*	1	0	1.42	0.03	2.1	19.7	4.0	20.3	CR
		f	67	2	0	0	2.11	0.82	38.9	51.2	21.1	41.2	CR
		f	32	-*	2	0	3.89	1.36	35.0	63.9	30.8	48.2	PD
	5	f	28	6	1	0	5.44	1.27	23.3	51.3	25.2	49.1	CR
		m	63	5	1	0	3.37	1.25	37.1	156.7	54.9	35.0	CR
		f	32	2	1	0	2.61	1.23	47.1	134.1	68.0	50.7	CR
	20	m	46	7	1	0	5.90	2.18	36.9	84.3	28.7	34.0	CR
		f	68	3	1	0	4.51	1.53	33.9	100.6	47.0	46.7	CR
		f	37	4	0	0	6.22	1.17	18.8	84.7	53.9	63.6	PD
	50	m	37	4	1	0	3.32	0.4	12.0	91.7	8.1	8.8	SD
		m	66	2	0	0	4.14	0.21	5.1	85.7	16.6	19.4	PD
		m	32	3	1	0	3.87	1.24	32	171.3	65.2	38.1	CR
		m	38	5	1	0	3.81	1.49	39.1	163.0	83.3	51.1	CR
	100	f	64	3	0	0	6.20	2.02	32.6	36.2	19.4	53.6	CR
		f	77	3	0	0	3.33	0.75	22.5	65.0	24.3	37.4	NE
		m	37	6	1	0	4.91	0.60	12.2	139.6	33.1	23.7	CR
	200	f	46	3	0	0	8.83	1.62	18.3	257.4	150.4	58.4	-*
	NI	m	47	3	0	0	3.17	0.41	12.9	61.1	10.1	16.5	-*
CLL	1	m	55	11	1	0	2.85	0.43	15.1	80.8	23.5	29.1	PR
	5	m	60	7	1	0	3.11	1.56	50.2	78.3	49.8	63.6	PR

	100	m	62	5	0	0	4.49	0.58	12.9	80.6	25.4	31.5	CR
		m	64	5	0	0	7.41	1.42	19.2	127.1	38.9	30.6	CR
	200	f	56	7	1	0	2.36	0.42	17.8	57.3	36.7	64.0	CR
		f	46	6	1	1	4.91	0.93	18.9	101.5	39.6	39.0	PR
		m	68	5	0	0	3.29	1.09	33.1	181.3	67.2	37.1	CR
		f	64	2	0	0	5.24	1.49	28.4	146.4	99.7	68.1	CR
	NI	m	52	5	0	0	1.35	0.49	36.3	14.8	6.4	43.2	-*
MCL	1	m	71	8	2	1	8.25	1.42	17.2	127.7	37.0	29.0	SD
	20	m	59	4	1	0	2.48	0.99	39.9	89.4	49.9	55.8	CR
		m	64	3	0	1	4.50	1.81	40.2	168.7	106.6	63.2	CR
	50	m	61	4	0	1	3.73	0.95	25.5	72.1	26.1	36.2	SD
	NI	m	71	3	0	0	13.64	4.50	33	169.8	42.2	24.9	-*
FL	1	f	72	9	0	0	2.1	0.97	46.2	54.9	36.8	67.0	PD
	20	m	48	6	0	0	8.08	2.00	24.8	241.3	131.4	54.5	CR
	50	m	75	4	0	0	6.63	1.98	29.9	126.4	56.9	45.0	CR
DLBCL	5	f	53	5	0	1	4.38	0.26	5.9	51.9	5.6	10.8	PD
		m	43	2	0	0	3.12	0.76	24.4	119.0	61.7	51.8	PD
	20	f	47	4	1	0	2.78	0.36	12.9	51.0	11.9	23.3	CR
		f	59	7	0	1	3.02	0.33	10.9	60.2	17.0	28.2	SD
	50	m	68	5	1	0	5.56	1.56	28.1	169.9	114.0	67.1	PD
	100	m	58	5	0	0	6.10	0.49	8	74.4	16.7	22.4	SD
	200	f	59	6	0	1	3.17	1.78	56.2	107.6	72.2	67.1	PR
B-PLL	200	m	46	6	1	0	3.79	0.99	26.1	169.9	100.3	59	PD

Abbreviations: ALL, acute lymphocytic leukemia; allo-SCT, allogenic stem cell transplantation; auto-SCT, autologous stem cell transplantation; B-PLL, B-cell prolymphocytic leukemia; BSA, body surface area; CLL, chronic lymphocytic leukemia; CR, complete response; DLBCL, diffuse large B-cell lymphoma; f, female; FL, follicular lymphoma; m, male; MCL, mantle cell lymphoma; NE, not evaluated; NI, no infusion; PD, progressive disease; PR, partial response; SD, stable disease; TNC, total nucleated cells; WBC, white blood cells; *no data available

Suppl. Table 3. Reasons for second apheresis

Reason for second apheresis	n	Diagnosis	CAR-T cell product
Target $CD3^+$ T-cell yield not reached	3	DLBCL	Tisa-cel
Low $CD3^+$ T-cell yield	1	DLBCL	Axi-cel
Contamination during apheresis - bacteria	2	DLBCL	Axi-cel
Nonconforming CAR T-cell product - particles	2	DLBCL	Axi-cel
Nonconforming CAR T-cell product – low CAR T-cell activity	1	CLL	HD-CAR-19
Nonconforming CAR T-cell product – low CAR T-cell amount	1	CLL	HD-CAR-19
Defective weld seam at apheresis product	1	DLBCL	Axi-cel
Apheresis not processed due to HBV infection	1	DLBCL	Tisa-cel

Abbreviations: CLL, chronic lymphocytic leukemia; DLBCL, diffuse large B-cell lymphoma; HBV, hepatitis B virus

Suppl. Table 4. Deaths Following CAR T-cell Therapy

Cause of Death	Days after CAR T-cell therapy	Gender	Age	Disease	Prior Lines of therapy	Prior SCT	Product	CRS Grade	ICANS Grade
Circulatory Shock	10	m	60	DLBCL	5	-	Axi-cel	3	4
Sepsis	41	m	72	DLBCL	3	-	Axi-cel	4	2
Sepsis	27	m	77	DLBCL	5	Auto-SCT	Tisa-cel	3	4

Abbreviations: Auto-SCT, autologous stem cell transplantation; CRS, Cytokine Release Syndrome; DLBCL, diffuse large B-cell lymphoma ICANS, Immune Effector Cell–Associated Neurotoxicity Syndrome; m, male; Tx, therapy
